# Employee Preference and Use of Employee Mental Health Programs: Mixed Methods Study

**DOI:** 10.2196/65750

**Published:** 2025-05-05

**Authors:** Benedict Sevov, Robin Huettemann, Maximillian Zinner, Sven Meister, Leonard Fehring

**Affiliations:** 1 Faculty of Health School of Medicine Witten/Herdecke University Witten Germany; 2 Health Care Informatics, Faculty of Health School of Medicine Witten/Herdecke University Witten Germany; 3 Department Healthcare Fraunhofer Institute for Software and Systems Engineering ISST Dortmund Germany; 4 Department of Gastroenterology Helios University Hospital Wuppertal, Witten/Herdecke University Wuppertal Germany

**Keywords:** digital health, mental health, workplace, employee well-being, employee mental health programs, intention to use, facilitators and barriers, mixed methods

## Abstract

**Background:**

Mental health issues represent a prevalent challenge for employees and their employers, leading to substantial impacts on individuals, society, and the economy. Different employee mental health programs (EMHPs) can support employees in preventing and treating mental health issues. However, the impact of such EMHPs depends largely on the use behavior of employees.

**Objective:**

This study aimed to gain deeper insights into employees’ attitude and use behavior regarding EMHPs by investigating (1) employee preference and intention to use EMHPs, (2) factors that predict use, and (3) key facilitators and barriers influencing use.

**Methods:**

An exploratory sequential mixed methods approach was applied, including a scoping review, qualitative interviews, and a quantitative web-based survey. Semistructured qualitative interviews were conducted with 15 employees to gain insights into EMHPs from the employee perspective and inform the creation of a web-based questionnaire. The quantitative web-based survey was conducted to collect representative primary data on employees’ perspectives on different EMHPs using 7-point Likert scales. The collected quantitative data were analyzed through descriptive and inferential statistics, including repeated measures ANOVAs and chi-square tests.

**Results:**

The final sample of the web-based survey consisted of 1134 participants and was representative across several sociodemographic characteristics. Analysis of the sample revealed that when given the choice, employees preferred digital (n=666, 58.73%) and self-intervention (n=590, 52.03%) EMHPs. Employees were most likely to use EMHPs focused on prevention (mean 4.89, SD 1.61). Intention to use EMHPs was predicted by age (young: mean 4.59, SD 1.2; old: mean 4.19, SD 1.4; *P*<.001; Cohen *d*=0.32), education (academic degree: mean 4.68, SD 1.24; no academic degree: mean 4.26, SD 1.32; *P*<.001; Cohen *d*=0.32), and mostly by company culture (positive company culture: mean 4.61, SD 1.27; negative company culture: mean 3.99, SD 1.27; *P*<.001; Cohen *d*=0.49). Cost coverage (n=345, 30.42%) and ease of use (n=337, 29.72%) were critical facilitators of use.

**Conclusions:**

Employers can have a positive contribution to employee mental health by starting to offer EMHPs, preferably digital self-intervention programs for prevention; creating and maintaining the right work environment and culture; and ensuring cost coverage for the EMHP.

## Introduction

### Background

Mental health issues represent a rising global challenge. In this paper, we broadly define mental health issues as any condition affecting an individual’s mental health. This definition aligns with the World Health Organization’s (WHO’s) term “mental health condition” and includes mental disorders as defined by the WHO [1]. According to the WHO, approximately 12% of the global population experience a mental health condition, and every fourth family has a member who is affected by a mental disorder [1,2]. Most of these individuals are employed and therefore represent the active workforce [3]. Data suggest that approximately 20% of employees in industrialized countries experience mental health issues, either through mild symptoms or more serious forms of mental disorders, with prevalence continuing to rise [3,4]. This leads to 3 main adverse effects. First, at the individual level, mental health issues deteriorate the mental health and well-being of employees and negatively impact their quality of life and job performance. Second, at the societal level, mental health issues place a significant burden on welfare systems, which must cover the high costs of treatment and pension requirements. Third, at the economic level, damage arises from decreased productivity, which causes a shrinking economic surplus for individual entities and the respective national economies [1,5,6]. Total annual costs of mental disorders, including health care expenses, costs due to lost productivity, and related economic impacts, are estimated to range between €100 billion and €150 billion (approximately US $110-$170) for countries such as Germany and France [7,8]. On a global scale, these costs amount to approximately US $5 trillion [9].

The workplace itself offers a promising environment for promoting good mental health by preventing mental health issues in early stages or mitigating the effects of existing mental disorders [10-12]. Many programs exist that are explicitly designed or used to support employees [13-16]. As no uniform umbrella term is commonly used for such programs, we refer to these programs as employee mental health programs (EMHPs). In the style of the broader definition of employee assistance programs (EAPs) of the Employee Assistance Professionals Association [17,18], we define EMHPs as any programs or solutions offered and facilitated by employers to support employees in sustaining or regaining good mental health. We consider all solutions independent of whether they stem from company internal units or external service providers. Real examples are manifold as employers offer their employees a diverse range of EMHPs, with the programs coming in different forms and formats [12,18-20]. Among others, there are 3 key dimensions to characterize EMHPs. First, the medium, that is, whether an EMHP is in a digital, analog, or hybrid form. Second, the interaction form, that is, whether an EMHP is self-guided, delivered in a one-on-one setting, or used in a group. Third, the addressed stage of mental health, that is, whether an EMHP is supposed to support employees in preventing mental challenges, treating existing mental disorders, or rehabilitating from past mental health issues [21-24]. Among EMHPs, digital programs are particularly popular and increasingly offered due to their advantages, such as cost efficiency, scalability, and flexibility in terms of time and location [25].

### Prior Work

Several scientific studies and meta-analyses have researched the effectiveness of EMHPs. While some studies have not found clear evidence for the effectiveness of EMHPs in improving mental health outcomes [26,27], many other studies suggest that EMPHs can have significant positive effects on employees’ mental well-being and thereby improve resilience and work effectiveness [15,18,28,29]. In recent years, studies and meta-analyses researching the effectiveness of digital EMHPs have gained particular attention and provide similar evidence for the potential positive effects of digital programs [20,25,30-33]. These positive effects have also been examined and found for different employee groups, including those in specific professions and across various countries [34-39]. Although both digital and analog EMHPs have been found effective in several studies, researchers note that the impact of these programs depends on the type of EMHP and the specific employee group they target [32,33,40].

Several studies have also identified different general facilitators of and barriers to using EMHPs [24,41,42]. However, only some studies have looked at differences in preference and use behavior based on employee-related characteristics, such as age and gender [43,44], type of profession [42], and education and income [45]. Existing research on predictive characteristics primarily focuses on specific EMHPs and select employee characteristics, leaving a gap in the literature regarding a comprehensive, holistic perspective on how different employee groups prefer and use various types of EMHPs. However, such a holistic view is essential to create transparency on which factors influence EMHP preference and use, particularly for digital ones. A deeper understanding of these predictors would benefit both researchers and practitioners. For researchers, it would help ensure that future research adopts an employee-centered approach. For employers and other stakeholders, it would help provide valuable insights into which EMHPs are most relevant and effective for their targeted workforce, ultimately increasing adoption rates. Greater adoption of EMHPs is likely to improve employees’ mental well-being and reduce symptoms, while also mitigating productivity losses. The value of and need for further research to determine suitable mental health resources for employees is also explicitly mentioned by researchers [46]. To the best of our knowledge, no comprehensive study exists that combines research on multiple predictive factors for the use of different EMHP types and relevant facilitators and barriers.

### Objectives

Therefore, the objective of this study was to address this research gap by (1) researching preference and intention to use of different types of EMPHs from the employee perspective, comparing digital EMHPs to nondigital ones; (2) examining several predicting factors for potential use of EMHPs; and (3) identifying key facilitators of and barriers to using EMHPs (research questions [RQs] 1-3).

On the basis of the first indications from existing research on EMHPs, as well as on initial evidence from research on the effects of several demographic, sociocultural, program, and other characteristics on intention to use or participation in general health interventions [47,48], we hypothesize that several employee-related factors, such as demographics and personal characteristics, and company-related factors, such as company culture, represent solid predictors for the use of EMHPs. As initial studies have observed different personal preferences for EMHPs among distinct demographic groups [43,44], the predictive value of the several considered characteristics may differ by type of EMHP, especially between digital and nondigital ones.

## Methods

### Exploratory Sequential Mixed Methods Approach

We applied an exploratory sequential mixed methods approach with 3 steps. The methodology and results of each step are reported separately, in line with the Mixed Methods Article Reporting Standards. The first step represented a scoping review to identify relevant research on EMHPs. The second step was an exploratory interview study for primary data collection. We conducted semistructured qualitative interviews to collect additional insights into the research topic and illustrate employees’ views on EMHPs. The third step was a quantitative web-based survey for primary data collection. For the survey, a closed-format questionnaire was created and validated based on the results of the previous 2 steps.

### Ethical Considerations

The Ethics Committee of Witten/Herdecke University raised no ethical concerns with regard to this research project (S-12/2023). All individuals who participated in either the qualitative interview study or the quantitative web-based survey provided voluntary, informed, and written consent to participate in the study and have the results published in a peer-reviewed article. The primary data collection approaches adhered to established ethical standards in scientific research as well as data protection regulations (General Data Protection Regulation). The data collected during the qualitative interview study were factually anonymized in a way that made it impossible, or only possible with disproportionately great effort, to identify individual participants. The data collected through the web-based survey were anonymized using the k-anonymity technique, which ensured that responses were removed if they did not meet the defined anonymity criteria. Additionally, demographic data obtained from the web-based survey such as age were collected in predefined intervals rather than as exact values to further ensure participant anonymity. The participants of the web-based survey received a monetary compensation from the research panel provider that executed the data collection. The research panel provider was remunerated by the authors.

### Scoping Review

A scoping review according to the PRISMA-ScR (Preferred Reporting Items for Systematic Reviews and Meta-Analyses Extension for Scoping Reviews) guidelines was performed to scope the existing research on EMHPs; refer to the PRISMA-ScR checklist (Multimedia Appendix 1) for further details. The review was conducted in 2 streams with the following 2 search queries performed in the “PubMed” database on January 12, 2023:

1. ((mental health[MeSH Terms]) OR (psychology[MeSH Terms])) AND ((employee assistance program[MeSH Terms]) OR (employer intervention[MeSH Terms]) OR (workplace intervention[MeSH Terms])) OR ((mental health[MeSH Terms]) AND (workplace[mesh terms]) AND (health promotion[mesh terms]))
2. (mental health[Title/Abstract]) AND ((employee assistance program[Title/Abstract]) OR (employer intervention[Title/Abstract]) OR (workplace intervention[Title/Abstract]))

**Figure 1:**
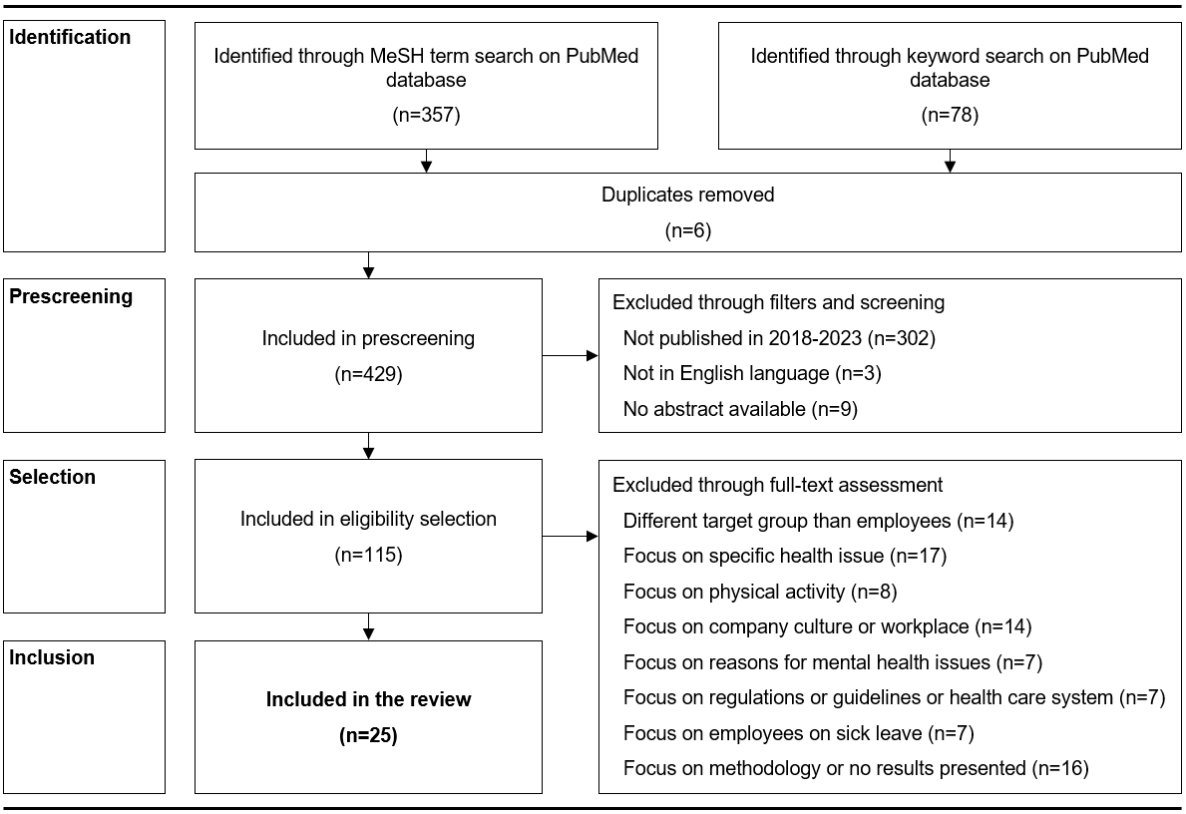
Overview of the scoping review process. The defined scoping review process included a systematic screening rationale according to the PRISMA-ScR (Preferred Reporting Items For Systematic Reviews And Meta-Analyses Extension for Scoping Reviews) guidelines, resulting in 25 literature records in the final review. MeSH: Medical Subject Headings.

The final set of literature records was identified based on a structured screening process; details are found in Figure 1. Backward and forward snowballing was then applied to reveal further relevant literature records. For snowballing, we allowed the inclusion of relevant records published outside the defined publication period used for the screening process. The overall identified literature was used to create the semistructured interview guide and the web-based questionnaire with its answer options (initial codes).

### Semistructured Exploratory Interview Study

The interview guide (Multimedia Appendix 2) was designed in an open-ended format to support the exploratory nature of the interviews and was structured along the defined RQs. The interview study aimed to identify the most relevant types of EMHPs, as well as all relevant facilitators and barriers from the employee perspective, to ensure that these were included in the web-based questionnaire. Results from the interviews were also used to illustrate relevant findings from the web-based survey, which, in turn, were interpreted in the context of the interview results. A purposive sampling approach was applied to obtain a representative participant sample of the observed population (employees of employers in Germany). The participants were recruited from the broader network of all authors and selected such that they were well distributed across the 5 demographic and company characteristics—age, gender, education, size of employer, and industry of employer. This ensured representativeness. All participants were informed in writing about the study’s purpose and data privacy terms. Selected parts of the resulting interview transcripts were analyzed and used for another research study.

The interviews were conducted in German, either in person or via phone or video call software, during February and March 2023. The interviews were recorded and transcribed in a factually anonymized way so that no participant or company names were stated, and personal data were categorized, for example, into age groups [49]. The transcripts were used to conduct qualitative data analysis through 2 approaches. First, we applied the directed content analysis approach outlined by Hsieh and Shannon [50] to identify the most relevant dimensions of EMHPs, for example, medium or interaction form. Second, we applied the thematic analysis approach described by Braun and Clarke [51] to identify relevant facilitators of and barriers to using EMHPs and relevant mental disorders.

The coding process was structured along 2 iterative coding cycles, and the coding was conducted and reviewed independently by 2 coauthors using the software MAXQDA (VERBI Software Consult Sozialforschung. GmbH) [52]. For both approaches, during the first coding cycle, single words or phrases were either matched with one of the initial codes derived from the scoping review or were defined as a new code. During the second coding cycle, the assigned codes were reviewed and further consolidated or separated depending on the thematic fit. The directed content analysis approach allowed for the prioritization of the most relevant dimensions of EMHPs from the employee perspective based on the frequency of the respective codes, which were limited to 1 per participant to avoid skewed data resulting from participants referring several times to 1 single code.

The COREQ (Consolidated Criteria for Reporting Qualitative Research) checklist describes the details of the applied method (Multimedia Appendix 3).

### Quantitative Web-Based Survey

#### Survey Design

The web-based questionnaire (Multimedia Appendix 4) was informed by the results of the qualitative interview study and designed in a closed-ended format. It was structured along the RQs with multiple- or single-choice questions and constructs based on established measures, assessed through 7-point Likert scales (except for mental health status). The questions and constructs represented predicting variables (PV), dependent variables (DV), or collected general information (GI).

#### Questions and Constructs

##### Demographic Characteristics (PV)

Data on age, gender, income, and education were collected through standard questions for such web-based surveys (categorical single-choice questions).

##### Digital Health Care Literacy (PV)

Participants rated their digital health care literacy using the Digital Health Care Literacy Scale by Nelson et al [53].

##### Mental Health Status (PV)

The current mental health status was assessed through a 3-item short form (Mental Health Inventory [MHI]; MHI-3) of the 5-item (MHI-5) measure [54]. To limit the overall number of questions, the MHI-3 was chosen for its brevity, while still being based on the well-established and widely used MHI-5 [55]. The items of the used MHI-3 were taken from a German version, and answers were collected through a 6-point Likert scale [56]. A mental health score was calculated and used to divide participants into 2 groups (positive or negative mental health status) based on a threshold defined by the scoring method of Rumpf et al [57]. Further, yes or no questions were asked to gain more detail on personal mental health status.

##### Relevance of Mental Health (GI)

The personal view on the relevance of mental health in society was assessed through a 1-item question. The mental disorders perceived as relevant among employees were assessed based on a multiple-choice question. The answer items were informed by literature and the interview study [58,59].

##### Company-Related and Profession-Related Characteristics (GI)

The number of employees, the industry of the employer, and the profession type were asked based on single-choice questions. Answer items were derived from established concepts and selectively adapted [60-62].

##### Company Culture (PV)

Data on company culture were collected through the German “Kurzskala zur Erfassung der Unternehmenskultur” (corporate culture scale, short form) by Jöns et al [63] using the 2 subscales leadership (5 items) and collaboration (4 items).

##### Intention to Use (DV)

Intention to use was measured as a DV for 9 different types of programs. The first 6 program types were derived from all combinations of the 2 dimensions of EMPHs: medium (digital and analog) and interaction form (self-intervention, bilateral intervention, and group intervention). The additional 3 program types were focused on the different addressed stages of mental health (prevention, treatment, and rehabilitation). All dimensions were clearly defined in the web-based questionnaire (Multimedia Appendix 4) to ensure a uniform understanding of all survey participants; for example, a program was defined as digital if it incorporated any form of digital technology, even if only a single component was digital. Intention to use was assessed with 1 item derived from the intention to use construct developed by Venkatesh et al [64].

##### Preference (DV)

To assess the unique preference for one of the first 6 programs, a single-choice question was asked.

##### Factors for Use (Facilitators and Barriers; DV)

Factors supporting and discouraging the use of EMHPs were assessed through 2 separate multiple-choice questions, each including an “Other” option with a free-text field. The given answer items were based on the scoping review and the qualitative interview study. The latter ensured the comprehensiveness of the selection of relevant facilitators and barriers from an employee perspective.

##### Offered EMHP (GI)

Participants were asked whether their employers offered any EMHP in the last 12 months and, if so, what type (medium, interaction form, and the addressed stage of mental health) and whether it was used. If participants used the program, their satisfaction was assessed through the 2-item system satisfaction construct by Wixom and Todd [65].

A control question was asked in the middle of the survey to check for participants’ attention.

#### Data Collection

The web-based survey was conducted in Germany in the German language in April and May 2023. A purposive sampling approach was applied to obtain a representative participant sample. Participant recruitment was conducted through a research panel provider that contacted registered panel participants from across Germany via email to invite them to participate in the study. As an inclusion criterion, participants needed to be employed by an employer in Germany for a minimum of 6 months to be able to talk about the mental health support at their current employer. Moreover, during open data collection, quotas were used to ensure a diverse sample with a representative distribution of the survey participants along the sociodemographic characteristics age, gender, and education. Official figures from the German Federal Statistics Office were used as reference values for the distributions to manage the quotas [66,67]. With this and the large sample size, it was ensured that the final sample was representative of the observed population of employees working for employers in Germany. Further details are provided according to the CHERRIES (Checklist for Reporting Results of Internet E-Surveys) checklist (Multimedia Appendix 5).

#### Descriptive and Inferential Statistical Analyses

**Figure 2:**
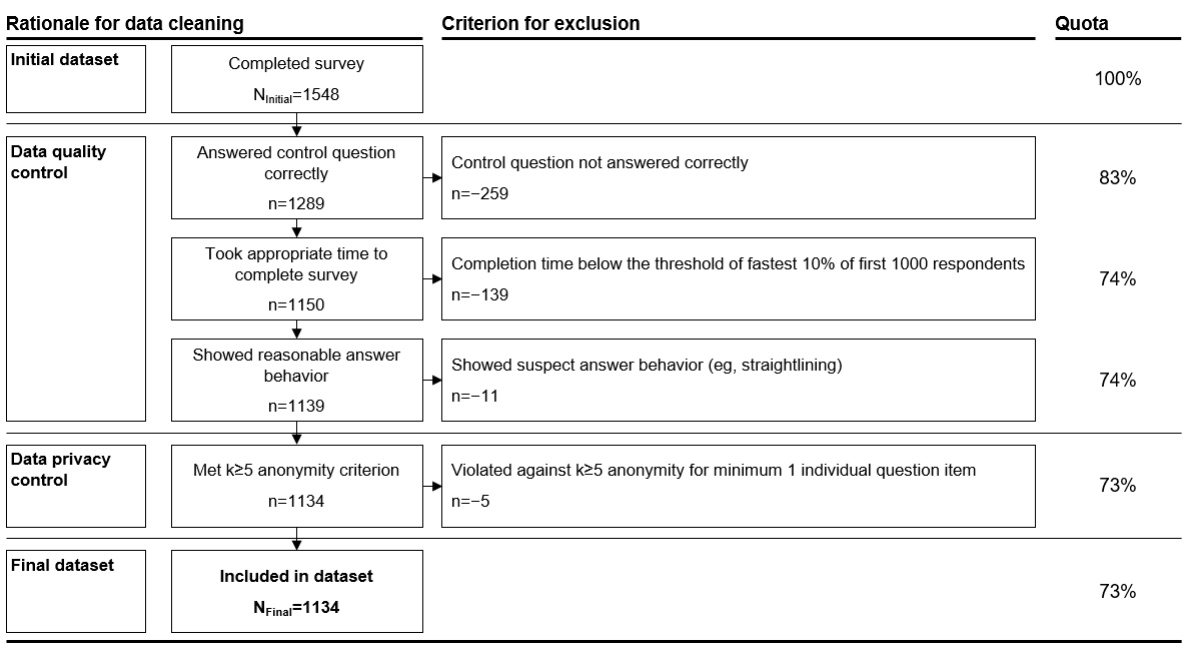
Overview of the data cleaning process. The defined data cleaning process included several data quality control steps and 1 data privacy control step to achieve the final dataset of 1134 participants. Suspect answer behavior means answers with strong patterns that might indicate careless responding, for example, straightlining.

After cleaning the survey data (Figure 2), multi-item constructs were tested for reliability using thresholds for Cronbach α based on the study by Blanz [68]. Subsequently, data were first analyzed using descriptive statistics. Second, data were analyzed using inferential statistical analyses. Chi-square tests were conducted to examine the relation between demographic characteristics and actual use of EMHPs and the relation between preference for specific EMHP types and perception of the relevance of influencing factors. Effect sizes were measured using phi (φ). Repeated measures ANOVAs were conducted to analyze the difference in intention to use different EMHPs of all participants (within-participants design) [69]. The Greenhouse-Geisser correction was applied when the assumption of sphericity was violated. The Bonferroni correction was applied for multiple comparisons. Effect sizes were measured using partial eta squared for the ANOVAs and Cohen *d* for the pairwise comparisons. One-way ANOVAs were conducted to identify differences in the means of the DV (intention to use) between different groups. Effect sizes of group differences were measured using Cohen *d*. Groups were formed based on relevant independent variables, such as demographics, mental health status (current or past), and company culture. Selected demographic variables were grouped into binary variables (eg, age into young vs old and education into nonacademic vs academic). Assuming interval scale level, the means of variables reported on Likert scales were calculated as arithmetic means [70]. The significance level was set at a *P* value of <.05. SPSS Statistics software (version 29.0; IBM Corp) was used for statistical analyses.

## Results

### Scoping Review

Through the scoping review, 25 records relevant to the characterization of EMHPs and factors impacting use of such programs were identified (Figure 1). Furthermore, 12 records relevant to the research topic were added through snowballing (an overview of all records can be found in Multimedia Appendix 6 [3-6,10-16,18-21,23-25,28-46]). A total of 45 initial codes for the web-based survey questionnaire were identified, including initial codes for relevant mental disorders.

### Semistructured Exploratory Interview Study

The final sample of the exploratory interview study comprised 15 participants. The interview study was concluded after reaching content saturation, which was indicated by recurring responses, confirmation of previously identified themes, and the absence of any new emerging themes [71]. The participants were well distributed across age groups, gender, education levels, industry, and company size (number of employees; Multimedia Appendix 7).

The qualitative interviews identified the most relevant dimensions of EMHPs from the employee perspective, including medium, interaction form, and the addressed stage of mental health. In total, 17 new codes were identified and added to the 45 initial codes derived from the scoping review. The most relevant facilitators mentioned by interview participants included employees needing to be aware of offered EMHPs and employers being willing to fully or partly pay for the offered program, each mentioned by 12 (80%) of the 15 participants. This was followed by the importance of support through culture and leadership, mentioned by 11 (73%) of the 15 participants. The most relevant barrier mentioned during the interviews was that using the EMPH would be too time-consuming, which would again increase pressure on employees willing to use it, mentioned by 10 (67%) of the 15 participants. This was followed by data protection and privacy concerns, mentioned by 9 (60%) of the 15 participants. As additional thoughts on the topic, 8 (53%) of the 15 participants, when being asked at the end of the interview, stated that they think it was a very critical topic and that more awareness across employers and employees would be needed.

### Quantitative Web-Based Survey

#### Data Sample

The final dataset of the web-based survey included 1134 responses after data cleaning (Figure 2). To the best of our knowledge, this represents the largest survey of employees in Germany in this research context and one of the largest globally.

As targeted by design, the final sample is representative of the German workforce across the relevant demographic characteristics age, gender, and educational degree [66,67], as well as distributed across the company-related characteristics number of employees, industry, and profession (Table 1).

**Table 1:** Distribution of responses in the final dataset across demographic and company-related characteristics (N=1134).

Variable			Participants, n (%)
**Age (years)**			
	≤29	214 (18.87)	
	30-39	236 (20.81)	
	40-49	222 (19.58)	
	50-59	291 (25.66)	
	≥60	170 (14.99)	
	Prefer not to answer	1 (0.09)	
**Gender**			
	Woman	523 (46.12)	
	Man	611 (53.88)	
	Nonbinary	0 (0)	
	Prefer not to answer	0 (0)	
**Education**			
	None	0 (0)	
	School degree except high school (“Abitur”)	79 (6.97)	
	High school degree (“Abitur”) or equivalent	108 (9.5)	
	Professional degree, vocational training, or equivalent	484 (42.68)	
	Bachelor’s degree or equivalent	201 (17.72)	
	Master’s degree or equivalent	215 (18.96)	
	Doctor or PhD degree or equivalent	43 (3.79)	
	Prefer not to answer	4 (0.35)	
**Number of employees**			
	≤9	99 (8.73)	
	10-49	199 (17.55)	
	50-249	261 (23.02)	
	250-499	140 (12.35)	
	500-999	142 (12.52)	
	1000-9999	161 (14.2)	
	≥10,000	132 (11.64)	
**Industry**			
	Energy and utilities	29 (2.56)	
	Raw materials and natural products	15 (1.32)	
	Industrial goods and services	170 (14.99)	
	Consumer goods and services	153 (13.49)	
	Commercial, technical, and scientific services as well as creative services	104 (9.17)	
	Health care	109 (9.61)	
	Financials and real estate	76 (6.7)	
	IT and communications	95 (8.38)	
	Public and governmental	125 (11.02)	
	Association, federation, and foundation	30 (2.65)	
	Other	228 (20.11)	
**Profession**			
	Governmental professionals, including the armed forces	72 (6.35)	
	Scientists and Researchers	34 (3)	
	Commercial professions, office professionals, and equivalent	370 (32.63)	
	Technicians and equivalent	140 (12.35)	
	Service-related professions (eg, restaurant and hotel) and sales workers	132 (11.64)	
	Social, teaching, and caring professions	119 (10.49)	
	Creative and art-related professions	30 (2.65)	
	Craft and related trades workers as well as machinery operators and assemblers	83 (7.32)	
	Skilled agricultural, forestry, and fishery workers	15 (1.32)	
	Other	139 (12.26)	

#### Measured Constructs

The 4 established constructs used were statistically reliable and internally consistent (Multimedia Appendix 8 [53,54,63,65]) [68]. The company culture measure showed excellent reliability, with a Cronbach α of 0.94 (9 items). The Digital Health Care Literacy Scale showed good reliability (Cronbach α=0.84; 3 items). The MHI-3 showed acceptable reliability (Cronbach α=0.76; 3 items). The EMHP satisfaction construct showed questionable reliability (Cronbach α=0.68; 2 items); however, given that Cronbach α generally is profoundly affected by the number of items and this construct only consists of 2 items, it can still be accepted as reliable [72].

#### Characteristics of the Sample and GI

MHI-3 suggested that 42.24% (471/1115) of employees had a negative mental health status. Independent of this, 52.84% (585/1107) of employees stated that they had issues with their mental health in the past. In total, 28.61% (321/1122) of employees stated that they pursue activities related to mental health, for example, psychotherapy, meditation, and using a mental health app. The mental health issues perceived as most relevant among employees were burnout (747/1134, 65.87% of participants), depression (717/1134, 63.23%), sleep disorders (704/1134, 62.08%), concentration issues (538/1134, 47.44%), and substance abuse or addiction (344/1134, 30.34%). Overall, employees rated the relevance of mental health in society as high (average score of 5.58, SD 1.56 on a 7-point Likert scale with 7=very relevant; median score of 6, IQR 5-7; 702/1134, 61.9% stated that mental health was “relevant” or “very relevant”).

#### Availability of EMHPs

Overall, 20.37% (231/1134) of participants mentioned that their employers offered at least 1 EMHP. Large companies were more likely to offer a program (117/435, 26.9% vs 30/298, 10.1% at smaller companies). Of the offered EMHPs, 65.8% (152/231) were digitally enabled, while 31.6% (73/231) were analog (6/231, 2.6% of participants did not know). Programs were equally distributed across interaction forms, with 33.3% (77/231) of them being self-intervention, 32% (74/231) bilateral intervention, and 28.6% (66/231) group intervention (14/231, 6.1% did not know). In total, 60.2% (139/231) of the offered EMHPs focused on prevention, 23.4% (54/231) on treatment, and 10% (23/231) on rehabilitation (15/231, 6.5% did not know; Multimedia Appendix 9).

#### Actual Use of EMHPs

In total, 54.8% (126/230) of employees with an EMHP available through their employer stated that they use it or have used it during the last 12 months. The use rate was higher when digital EMHPs were offered compared to when analog EMHPs were offered (digital: 94/152, 61.8%; analog: 32/72, 44.4%; *P*=.001; φ=0.24; Multimedia Appendix 10). Younger employees (<50 years) had a higher use rate compared to older ones (≥50 years; young: 107/174, 61.5%; old: 19/56, 34%; *P*<.001; φ=0.24; Multimedia Appendix 11). Employees with an academic degree (Bachelor’s, Master’s, or Doctor or PhD degree) had a higher use rate compared to employees without an academic degree (academic degree: 93/139, 66.9%; no academic degree: 33/91, 36%; *P*<.001; φ=0.30; Multimedia Appendix 12). Employees who used offered EMHPs stated a generally high satisfaction with the used program (average satisfaction score of 5.6, SD 1.2 on a 7-point Likert scale; median score of 6.0, IQR 5.0-6.5; 70/126, 55.6% of employees reported a score of ≥6), while there is no significant difference in satisfaction of employees using digital versus analog programs.

#### Preferred EMHPs

When stating their unique preference, 58.73% (666/1134) of employees stated their preference for digitally enabled EMHPs and 52.03% (590/1134) for self-intervention EMHPs. The most preferred EMHP combination of medium and interaction form was the digital self-intervention program (371/1134, 32.72% of participants), followed by the analog self-intervention program (219/1134, 19.31%) and the digital bilateral intervention program (152/1134, 13.4%), which represents part 1 of RQ1.

#### Intention to Use EMHPs

**Figure 3:**
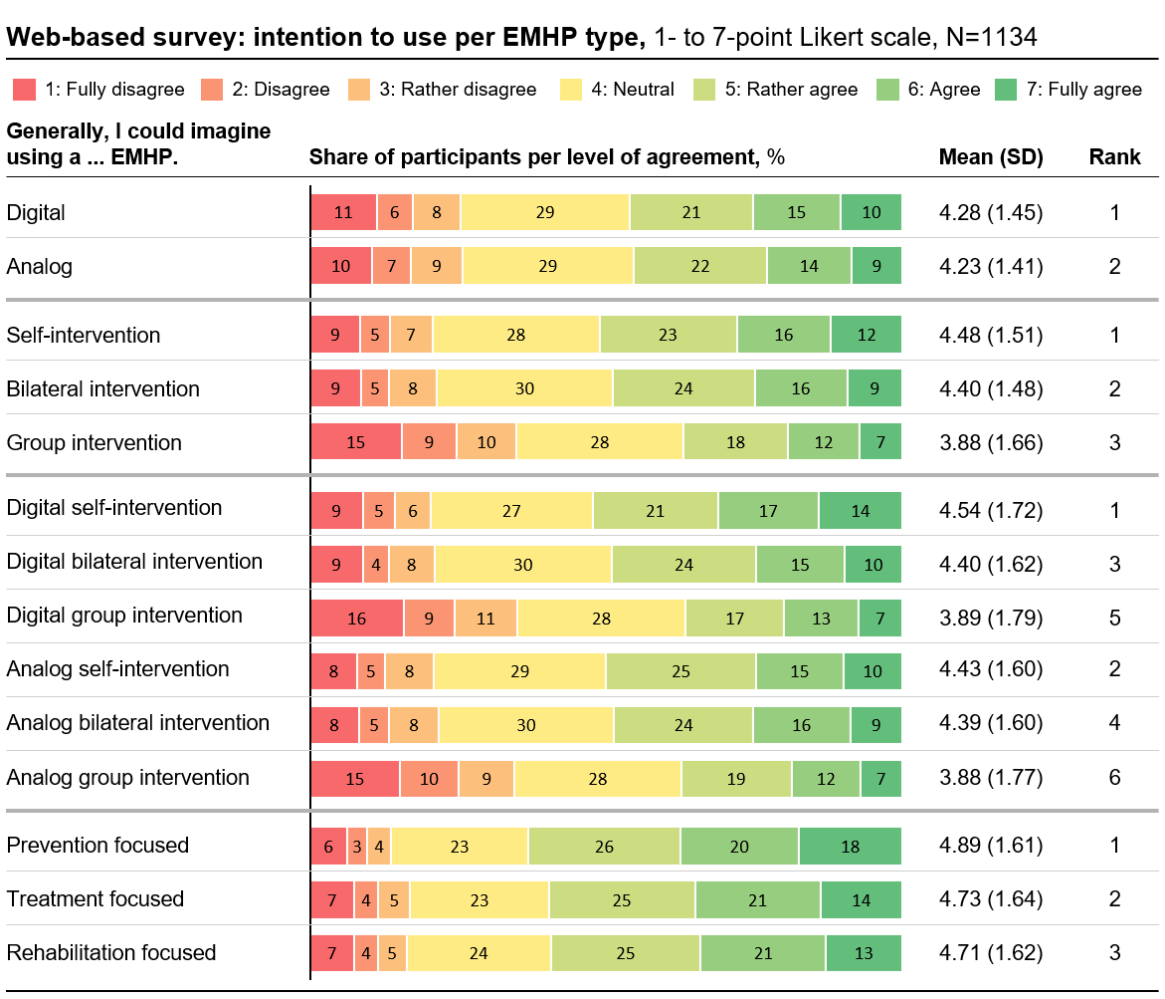
Results of the web-based survey on intention to use per employee mental health program (EMHP) type. Intention to use was assessed by asking the concrete intention to use on a 7-point Likert scale for the 6 EMHP types resulting from the combination of the dimensions medium (ie, digital and analog) and interaction form (ie, self-intervention, bilateral intervention, and group intervention), as well as for the 3 EMHP types based on the addressed stage of mental health (ie, prevention, treatment, and rehabilitation). The reported intention to use each EMHP type by medium and interaction form was calculated by aggregating the intention-to-use data for the 6 combined EMHP types.

Overall, there was medium intention to use EMHPs, with an average of 4.43 on a 7-point Likert scale (median 4.56, IQR 3.89-5.22). However, the SD was high, with an SD of 1.30 (variance 1.68). On average, 50.88% (577/1134) of participants indicated a positive intention to use an EMHP (average score of >4.5 on a 7-point Likert scale), while 31.57% (358/1134) were neutral (average score of >3.5 and <4.5). Regarding part 2 of RQ1, there was no significant difference between the overall intention to use digital and analog programs when they were available. However, intention to use was significantly higher for self-intervention programs and bilateral intervention programs (self: mean 4.48, SD 1.51; bilateral: mean 4.40, SD 1.48) compared to group intervention programs (group: mean 3.88, SD 1.66; *P*<.001 each; Cohen *d*=0.40 and Cohen *d*=0.39, respectively). The difference in intention to use between self-intervention and bilateral intervention programs was also significant (*P*=.03; Cohen *d*=0.08). Considering both dimensions, medium and interaction form, intention to use was highest for a digital self-intervention program (digital self-intervention: mean 4.54, SD 1.72; 593/1134, 52.29% of participants indicated a positive intention to use, ie, a score of ≥5). Intention to use for EMHPs focusing on prevention was significantly higher than that for EMHPs focusing on treatment (prevention: mean 4.89, SD 1.61; treatment: mean 4.73, SD 1.64; *P*<.001; Cohen *d*=0.13) and that for EMHPs focusing on rehabilitation (rehabilitation: mean 4.71, SD 1.62; *P*<.001; Cohen *d*=0.14). Refer to Figure 3 for an overview of the intention-to-use results (details of all repeated measures ANOVAs can be found in Multimedia Appendix 13).

#### Predictors for the Use of EMHPs

Addressing RQ2, it was found that intention to use was higher for younger employees (young: mean 4.59, SD 1.2; old: mean 4.19, SD 1.4; *P*<.001; Cohen *d*=0.32). No significant difference in overall intention to use nor regarding digital or analog EMHPs could be observed between women and men. However, women showed a higher intention to use self-intervention programs (women: mean 4.61, SD 1.49; men: mean 4.38, SD 1.52; *P*=.008; Cohen *d*=0.16) as well as prevention and treatment programs (prevention: mean_women_ 5.06, SD 1.51 and mean_men_ 4.74, SD 1.68; treatment: mean_women_ 4.90, SD 1.58 and mean_men_ 4.57, SD 1.68; *P*<.001 each; Cohen *d*=0.20 each). Intention to use was higher for employees who held academic degrees (Bachelor’s, Master’s, or Doctor or PhD degree; academic degree: mean 4.68, SD 1.24; no academic degree: mean 4.26, SD 1.32; *P*<.001; Cohen *d*=0.32). Given that digital health care literacy was high across the whole sample (average digital health care literacy score of 6.03, SD 1.18 on a 7-point Likert scale; median score of 6.33, IQR 5.33-7.00; 774/1134, 68.25% of employees reported a score of ≥6), further analyses did not reveal additional insights. Intention to use was higher for employees who had faced mental health issues in the past (mental health issues in the past: mean 4.64, SD 1.14; no mental health issues in the past: mean 4.22, SD 1.41; *P*<.001; Cohen *d*=0.32) and for employees with a negative self-assessed mental health status according to MHI-3 (positive mental health status: mean 4.31, SD 1.33; negative mental health status: mean 4.56, SD 1.21; *P*=.002; Cohen *d*=−0.19). Employees who perceived their company culture as positive reported a higher intention to use EMHPs (positive company culture: mean 4.61, SD 1.27; negative company culture: mean 3.99, SD 1.27; *P*<.001; Cohen *d*=0.49; details of all ANOVAs can be found in Multimedia Appendix 14).

#### Facilitators of and Barriers to the Use of EMHPs

The most relevant factor for employees impacting the use of EMHPs is whether the employer paid for the program (345/1134, 30.42%). This top facilitator was significantly more relevant for employees who preferred analog EMHPs (160/468, 34.19%) versus those who preferred digital EMHPs (185/666, 27.78%; *P*=.02; φ=−0.07). In line with this, employers not paying for the EMHP was the number 1 barrier (433/1134, 38.18%). Ease of use was the second most relevant facilitator (337/1134, 29.72%), with no difference between employees preferring digital or analog EMHPs. Content quality was another important facilitator if given (259/1134, 22.84%) and an even more relevant barrier if not given (301/1134, 26.54%). The top 5 facilitators and barriers are shown in Table 2, representing RQ3 (details of all chi-square tests can be found in Multimedia Appendix 15).

**Table 2:** Top 5 facilitators of and barriers to using employee mental health programs (EMHPs) by total participants and differences by preference for medium (N=1134).

	Total participants^a^ (N=1134), n (%)	Participants preferring digital EMHPs^b^ (n=666), n (%)	Participants preferring analog EMHPs^b^ (n=468), n (%)	*P* value^c^
**Top 5 facilitators**				
1. EMHP is paid for by the employer	345 (30.42)	185 (27.78)	160 (34.19)	.02
2. EMHP is easy to use	337 (29.72)	198 (29.73)	139 (29.7)	.99
3. EMHP with trustworthy data protection	282 (24.87)	168 (25.23)	114 (24.36)	.74
4. EMHP with easy access	265 (23.37)	153 (22.97)	112 (23.93)	.71
5. EMHP with high content quality	259 (22.84)	164 (24.62)	95 (20.3)	.09
**Top 5 barriers**				
1. EMHP requires full payment or copayment by the employee	433 (38.18)	250 (37.54)	183 (39.1)	.59
2. EMHP requires too much time	326 (28.75)	202 (30.33)	124 (26.5)	.16
3. EMHP with low content quality	301 (26.54)	173 (25.98)	128 (27.35)	.61
4. Employees feel forced to use EMHP	294 (25.93)	154 (23.12)	140 (29.91)	.01
5. EMHP is too complicated or requires too much effort	265 (23.37)	152 (22.82)	113 (24.15)	.60

^a^Share of total participants perceiving the facilitator or barrier as relevant.

^b^Share of participants preferring digital or analog EMHPs perceiving the facilitator or barrier as relevant.

^c^Significance level of the differences between participant groups preferring digital or analog EMHPs based on *P* values according to the chi-square test.

## Discussion

### Principal Findings

#### Generalizability of Findings

This study analyzed the deployment and use of EMHPs at companies across industries; the preference and intention to use of employees for different EMHPs, including relevant predictors; as well as facilitators and barriers. The representative and exceptionally large sample of the German working population allows for generalization of the findings. Generalizability is enhanced through the fact that no specific sociodemographic subgroup but a broad representative sample of the observed population across several sociodemographic characteristics was investigated. The representative distribution of education levels in the sample further ensured that no employees from a specific socioeconomic status group were underrepresented. The findings of the study are not only generalizable to Germany but may also be transferable to other countries, particularly industrialized nations where mental health challenges are comparable and employer-employee relationships tend to be similarly structured. Given that a relevant share of participants’ employers in this study were large organizations, many of these were likely to operate in several countries across the globe, where the general organizational culture and relationship between employer and employees can be expected to be similar. Moreover, the research on EMHPs generally focuses on the role of the employer as a provider of or at least an access point for mental health services. Therefore, this research is less dependent on the respective health system of a specific country but rather on universal predictors and factors of the relationship between employers and employees. To generate further insights into the transferability of the findings, this survey can be replicated in other industrialized countries by future research.

#### Prevalence and Actual Use of EMHPs

The fact that employees need support due to increasing mental health issues [16,28,31] is supported by our results, which illuminate the prevalence of past issues and current negative mental health status. While only a fifth of employees have access to EMHPs, more than half of these employees use them. This use rate seems to be high compared to numbers from other studies, indicating use rates for very specific psychotherapeutic consultation services and general EAPs of 1% up to 10% [73] or 39% when looking at intended use [44]. Hence, use rates seem to vary depending on the concrete EMHP. Given that the results of other studies focus on EAPs or consultation services for primarily existing issues, the overall use rates for all EMHPs, including prevention, are expected to be higher, as indicated by our results. Further studies should focus on measured use rates for different types of EMHPs, including prevention-focused programs.

#### Preferred EMHPs

The found preference for digital programs (666/1134, 58.73% of participants) and for self-intervention programs (590/1134, 52.03%) when choosing one preferred EMHP is in line with the desire for time- and location-wise flexibility and for anonymous use concerning colleagues [24,41]. While patients with existing mental health issues realize the potential advantages of digital programs [74], prior research has found that they prefer face-to-face mental health interventions or hybrid models that include face-to-face contact, presumably due to higher perceived effectiveness [74-76]. However, these studies focused on interventions treating existing mental health issues, for which many do not think that digital programs can replace traditional in-person psychotherapy adequately [77-79]. Furthermore, these studies did not explicitly focus on EMHPs in the work context but on the general population. Given that our study focuses on EMHPs that often also cover prevention purposes [24], the findings do not contradict each other. Instead, they indicate that preference for an intervention involving a mental health professional is higher when facing concrete, particularly severe issues, which was supported by our qualitative interviews:

I believe, if my mental state is seriously endangered, then I need a personal conversation, personal support. But if my mental state is somehow not good but not severely bad, then I can imagine using an app for myself, which can help me to feel better.

Participant 9

For prevention, tips for exercises, general information, or apps, etc, are very important. When there is a concrete need for treatment, then a one-on-one is the best option, with a psychotherapist.

Participant 7

However, digital and self-intervention programs are well suited for prevention purposes, especially in the workplace context [44], and digital EMHPs can even be as effective as nondigital ones regarding positive mental health outcomes [30]. A recent study confirms a strong preference for both types of EMHPs, digital self-intervention programs and programs delivered face-to-face [80]. While we acknowledge that preference for a certain EMHP type does not necessarily mean that these preferred EMHPs have higher effectiveness in obtaining the desired outcome, the intrinsic interest to use a specific EMHP is a relevant factor to start using such a program. As digital and nondigital EMHPs can have significant positive mental health effects, starting to use such a program is the most relevant first step, and consequently, the preference should be considered. The importance of intrinsic motivation and fun for starting to use an EMHP was also mentioned during our qualitative interviews:

Ideally, using it means fun. The people need a motivation to use it.

Participant 9

It is an important factor that the UX design is nice and that it is fun.

Participant 11

It needs to have an entertaining value; something needs to happen. Otherwise, you don’t get the people to do it.

Participant 13

#### Intention to Use EMHPs

Overall, we found a medium intention to use EMHPs. However, the detailed results show a strong distribution, indicating opposing views on such programs. One part has a high intention to use both digital and analog EMHPs, while another part is very reluctant. Individuals with a high intention to use EMHPs may be influenced by the increasing awareness of mental health through more research and more coverage in mainstream media, which is also driven by celebrities raising awareness [81,82]. In contrast, other individuals neglect the need, as well as their eligibility, for such programs and for dealing with mental health more openly in general, partly driven by stigmatization, which could explain participants with low intention to use [28,83-85].

#### Predictors for Use of EMHPs

In line with previous research, intention to use differs substantially by several demographic and personal characteristics [44,45].

##### Age

Younger individuals tend to be more open toward EMHPs. This may be due to higher awareness and a more open mindset of younger generations toward mental illness, which is found in prior research [42,86]. Conversely, other research suggests that older age is a predictor of help-seeking behavior [87,88]. This calls out for further research about differences in age concerning awareness and behavior regarding mental health support. Our findings support that younger generations are more open toward the topic and are more likely to use digital programs.

##### Gender

In contrast to other identified predictors that align with previous research, in this study, we found no evidence for differences in overall intention to use between women and men. However, when looking at specific EMHP types, women showed a higher intention to use programs focused on prevention and on treatment. This suggests that women might have a higher intention to use when presented with the concrete objective of the EMHP. The higher preference of women for self-intervention programs resonates with the findings of Smail-Crevier et al [44], who found that women have a significantly higher preference for self-help interactive programs than men. Further studies focusing on specific EMHPs with clearly defined dimensions, for example, interaction form, are needed to assess differences in preference by gender and gain deeper and solid insights.

##### Education

Intention to use is also found to be higher for individuals with higher formal education, which is in line with what has been suggested by prior research [45]. This might be explained by higher awareness and understanding of the challenge of mental health issues for individuals and society.

##### Previous Mental Health Issues

We found that individuals who indicate having had mental health issues in the past show a higher intention to use EMHPs. This suggests that awareness, primarily through lived experience with some mental health issues, increases the likelihood of seeking help and using offered support. Previous research has also found that positive experience with help seeking in the past increases the likelihood of seeking help again [87], which might also be the case for participants of our study, explaining the measured effect.

##### Mental Health Status

Furthermore, we found that the intention to use is higher for people who have a negative self-reported mental health status. Overall, this seems evident, as the need to use an EMHP is plausibly higher for these individuals than for people with good mental health. However, existing literature offers contrary findings regarding the effect of existing mental health issues on using support services. Some research suggests that when mental health issues reach a certain level of severity, individuals may experience reduced motivation and effort to engage with programs or seek treatment [79]. This effect is especially observable in people dealing with depression [41]. Other studies, in contrast, indicate that support is rather sought when issues already affect social functioning [87,89]. Hence, the severity of a present mental disorder plays a significant role in the resulting use of support services such as EMHPs. Therefore, further research could focus on the intention to use EMHPs based on the severity of mental health issues.

##### Company Culture

Another predictor of intention to use found in this study is the company culture. Individuals indicating a positive company culture at their workplace have a significantly higher intention to use EMHPs. This finding resonates with existing research on the importance of company culture and the positive effect of supportive leaders [5,13,46]. Notably, company culture emerged as the strongest predictor of intention to use EMHPs in this study, surpassing the effects of both age and education. The authenticity of the employer toward the offered program and the high relevance of the company culture were also mentioned by participants during the qualitative interviews:

It is important to feel that the employer means it, that it is a truly honest offer.

Participant 1

I want to have the feeling that this is not a one-time initiative to boost the image. I want to see the true intention of the employer, shown by continuous actions.

Participant 11

I need to have trust that I do not have to fear any negative consequences when I use it.

Participant 9

It is important that you have an environment where people do not judge, where colleagues say, “It is alright to use it, there is nothing condemnable with it.”

Participant 3

#### Facilitators of and Barriers to the Use of EMHPs

Having the EMHP paid by the employer is the most relevant facilitator and represents the largest barrier if this is not the case. The reason that cost coverage is an even more important factor for employees preferring analog EMHPs might be that analog programs are usually more expensive than digital ones [25]. While the cost coverage factor is not explicitly mentioned in existing studies on facilitators and barriers, research suggests that it is in the nature of EMHPs to be offered to employees for free or at least at a low cost [42]. A potential expectation attitude of German citizens particularly might stem from the fact that they are used to reimbursed health care services, including psychotherapeutic treatment [76] and apps (specific digital health apps called DiGAs in Germany) [90]. In line with this, the qualitative interviews indicated that if the employer offers a program, employees expect financial support as a basic condition:

It needs to be paid by the employer, at least the largest share.

Participant 10

If it costs me personal money, this is a huge barrier.

Participant 3

If it needs to be paid fully or even partially by the employee, there is no more willingness to use it.

Participant 5

Further relevant factors are ease of use, easy access, use that should not require too much time, and quality of the content. Hence, convenience, that is, being able to integrate use into everyday life, and the quality of EMHPs are essential for employees, as is indicated by previous studies [14,24,41,91] and our qualitative interviews:

Use has to be simple and clearly explained.

Participant 7

Access to the program should be easy.

Participant 3

The time effort should be reasonable. I would like to have it easily integrated into my daily routines.

Participant 1

It not only has to have good content, it also has to be easily understandable and interesting.

Participant 14

Employers should ensure these criteria are met to increase the likelihood of use and enhance the impact of EMHPs. They should also consider digital EMHPs, which are generally more cost-efficient, easier to scale, and more flexible than nondigital programs [25].

### Limitations

Despite high research standards, this study has some limitations. First, given that quantitative data collection was based on a web-based survey, it is likely that the results overrepresent digitally literate participants [92]. Therefore, participants might have been more prone to using digital EMHPs [93]. However, only a web-based survey of this nature can provide such a large sample representative across several demographic characteristics.

Second, for simplicity, the medium of EMHPs was categorized into a binary set with digital and analog EMHPs, whereas in reality, hybrid forms are available. Despite the existence of hybrid forms, the chosen definition was appropriate for the scope and objectives of our research, and the definitions were clearly presented in the web-based survey.

Third, the survey results relied on self-report measures and question items and the participants’ chosen options, which may have led to some skewness in the data. For instance, participants might have indicated their intended and not their actual use behavior when asked, a phenomenon commonly referred to as the intention-behavior gap [94]. In addition, results on the available EMHPs were based on the participants’ perspectives and not necessarily on the factual availability of the EMHPs offered by their employers.

### Conclusions

Our study contributes to a deeper scientific understanding of employees’ perspectives on different EMHPs, including preference, intention to use, predictors for use, and facilitators and barriers. This deeper understanding is relevant for academia and occupational practice. On the basis of our research, academia can drive further scientific studies and findings. Employers who are willing to actively contribute to the mental health of their employees can leverage our findings to choose and facilitate EMHPs, while providers can leverage these findings to create relevant EMHPs that match the employees’ needs.

The 3 main implications of the study indicate simple but meaningful actions for employers worldwide. First, because a relevant portion of employees use EMHPs when available and most report satisfaction with them, it is a strong starting point to consider offering an EMHP. Initially, the type of EMHP is not of utmost importance. While starting with digital self-intervention programs focused on prevention may lead to greater acceptance and adoption, given overall preferences, employees generally appear to value the simple availability of any EMHP. Hence, offering any EMHP should potentially create a positive impact. Naturally, the more targeted and tailored the EMHP is, the higher the probability of positive effects. However ultimately, the most important step is to start offering an EMHP.

Second, employers can steer one of the most relevant factors predicting the use of EMHPs—the company culture. This predictor has significant impact on use across all EMHP types. Therefore, employers are advised to create an authentic strategy and communication on offering EMHPs and genuinely support the opportunity to use an EMHP. Conversely, offering an EMHP while having a negative company culture with demotivating communication, leadership, and teamwork styles will likely have no relevant positive effect.

Third, employers should ensure that employees seeking support or willing to act preventatively do not have to pay to use an offered EMHP. Furthermore, they should provide EMHPs that are easy to use, easy to access, and of good quality, as these are the key facilitators of use. Thus, employers should consider offering digital EMHPs, which typically address several of these relevant factors.

## Data Availability

The datasets generated or analyzed during this study are available from the corresponding author on reasonable request.

## Appendix

Multimedia Appendix 1 The 22-item PRISMA-ScR (Preferred Reporting Items for Systematic Reviews and Meta-Analyses Extension for Scoping Reviews) checklist.

Multimedia Appendix 2 Interview guide for the exploratory qualitative interview study.

Multimedia Appendix 3 The 32-item COREQ (Consolidated Criteria for Reporting Qualitative Research) checklist.

Multimedia Appendix 4 Web-based questionnaire for the quantitative web-based survey.

Multimedia Appendix 5 The 30-item CHERRIES (Checklist for Reporting Results of Internet E-Surveys) checklist.

Multimedia Appendix 6 Overview of the literature identified through scoping review and snowballing.

Multimedia Appendix 7 Demographic and company-related characteristics of participants in the interview study (N=15).

Multimedia Appendix 8 Reliability and factor analyses of the used constructs.

Multimedia Appendix 9 Distribution of different types of employee mental health programs offered by employers in Germany.

Multimedia Appendix 10 Cross-table of the chi-square test of independence for the relation between the medium of available employee mental health program and actual employee mental health program use.

Multimedia Appendix 11 Cross-table of the chi-square test of independence for the relation between the binary age variable and actual employee mental health program use.

Multimedia Appendix 12 Cross-table of the chi-square test of independence for the relation between the binary education variable and actual employee mental health program use.

Multimedia Appendix 13 Results of repeated measures ANOVAs analyzing differences in intention to use different employee mental health programs, differentiated by several dimensions.

Multimedia Appendix 14 Mean comparisons of intention to use different employee mental health programs between the 2 groups of each binary predicting variable using ANOVA.

Multimedia Appendix 15 Chi-square tests of independence for the relation between employee groups preferring digital or analog employee mental health programs and perceiving specific facilitators and barriers as relevant for employee mental health program use.

## Abbreviations

CHERRIES: Checklist for Reporting Results of Internet E-Surveys
COREQ: Consolidated Criteria for Reporting Qualitative Research
DV: dependent variable
EAP: employee assistance program
EMHP: employee mental health program
GI: general information
MHI: Mental Health Inventory
PRISMA-ScR: Preferred Reporting Items for Systematic Reviews and Meta-Analyses Extension for Scoping Reviews
PV: predicting variable
RQ: research question
WHO: World Health Organization
