# Supplementary material for: Employee Preference and Use of Employee Mental Health Programs: Mixed Methods Study
Source: JMIR Hum Factors. 2025 May 5;12:e65750. doi: 10.2196/65750 (PMC12089874; doi:10.2196/65750)
Supplement: Multimedia Appendix 1 [file humanfactors_v12i1e65750_app1.docx]

**Multimedia Appendix 1. The 22-item PRISMA-ScR (Preferred Reporting Items for Systematic Reviews and Meta-Analyses Extension for Scoping Reviews) checklist.**

| **Item** | **Description** | **Item reporting** |
| --- | --- | --- |
| **Section: Title** |  |  |
| 1. Title | Not applicable – scoping review not predominant methodology | - |
| **Section: Abstract** |  |  |
| 2. Structured summary | **Introduction:** Different employee mental health programs (EMHPs) are deployed to support employee mental health. Different types of EMHPs exist and different factors affect use of these programs.  **Objective:** The objective was to identify relevant literature to prioritize relevant dimensions of EMHPs and identify relevant factors for the use of EMPHs (facilitators and barriers) to inform the creation of the web-based questionnaire.  **Methods:** A scoping review was applied to identify relevant studies about EMHPs. The “PubMed” database was searched with 2 search queries. Eligibility criteria were defined around year of publication, language, abstract availability, and different content-related criteria (Figure 1). The review was complemented by backward and forward snowballing.  **Results:** 25 records of relevant literature were identified. From these and 12 further records identified through snowballing, the relevant dimensions of EMHPs and relevant facilitators and barriers were derived. Overall, 45 initial codes were identified, including initial codes for relevant mental disorders.  **Conclusion:** The scoping review was an adequate approach to identify relevant studies about the topic and derive the targeted insights for the creation of the web-based questionnaire. | Figure 1; Multimedia Appendix 6 |
| **Section: Introduction** |  |  |
| 3. Rationale | EMHPs are deployed to support employees in sustaining or regaining a good mental health status. Different types of EMHPs exist, describable across several different dimensions. Besides, many different facilitators and barriers affect the actual use of EMHPs. To gain new insights and a more granular view on the topic through new research, a web-based questionnaire for a large web-based survey was to be created. A scoping review seemed most appropriate to research and scope existing literature to be able to build the web-based survey on previous findings. Hence, the scoping review was embedded as a starting point of the overall mixed methods approach of the study. | Methods |
| 4. Objectives | The scoping review aimed at identifying and prioritizing relevant dimensions of EMHPs and identifying relevant facilitators of and barriers to using EMPHs found by existing research to inform the creation of the web-based questionnaire. | Methods |
| **Section: Methods** |  |  |
| 5. Protocol and registration | Not applicable | - |
| 6. Eligibility criteria | To be included in the scoping review, journal articles needed to be listed on the “PubMed” database. Unique articles from 2 search queries were included in the prescreening process, during which the articles were assessed based on filters and technical criteria. Articles published between January 1, 2018 and including January 11, 2023, in English language, and providing an abstract were considered for the subsequent eligibility selection process. The publication period of 5 years from 2018 to beginning 2023 was chosen to only include current research without too old studies given the high dynamics in this area. The reason for the start year 2018 was that 2 full years of research conducted prior to the Covid-19 pandemic should be considered. The remaining articles were then selected based on full-text assessment during the eligibility selection process based on specific content-related eligibility criteria (Figure 1). | Figure 1 |
| 7. Information sources | The scientific database “PubMed” was searched to conduct the scoping review. The search queries were performed on January 12, 2023. | Methods |
| 8. Search | The search was conducted by Benedict Sevov with defined search queries in 2 streams. First, combinations of defined MeSH terms were used to search the database:  (1) ((mental health[MeSH Terms]) OR (psychology[MeSH Terms])) AND ((employee assistance program[MeSH Terms]) OR (employer intervention[MeSH Terms]) OR (workplace intervention[MeSH Terms])) OR ((mental health[MeSH Terms]) AND (workplace[mesh terms]) AND (health promotion[mesh terms]))  Second, defined key words were searched for in the articles’ titles and abstracts:  (2) (mental health[Title/Abstract]) AND ((employee assistance program[Title/Abstract]) OR (employer intervention[Title/Abstract]) OR (workplace intervention[Title/Abstract])) | Methods |
| 9. Selection of sources of evidence | A 2-step screening rationale was applied to identify the relevant articles. First, the found articles were prescreened through filters and review of technical criteria regarding publication date, language, and existence of an abstract. Second, the remaining articles were selected through a full-text assessment based on the defined content-related eligibility criteria (Figure 1). | Figure 1 |
| 10. Data charting process | All included and snowballed articles were recorded in a tabular overview including title, DOI, authors, journal, year of publication, methodology, geographic scope, research focus and derived insights. The derived insights attributes indicate which findings were used to inform the creation of the web-based questionnaire. Stated types of EMHP were used to identify the most relevant ones while found facilitators and barriers were used to define the initial codes. The text of the articles was carefully screened and respective parts were coded to identify the initial codes. | Multimedia Appendix 6 |
| 11. Data items | First, all included and snowballed articles were screened for insights about different possible dimensions of EMHPs. EMHP dimensions were derived from the findings and prioritization was based on the frequency of appearance and relevance of different dimensions. Prioritization was finalized based on the results from the qualitative interviews. Second, all included and snowballed articles were screened for potential facilitators of and barriers to using EMHPs. The derived initial codes were used to inform the creation of the web-based questionnaire. All initial codes were complemented by new codes derived from the results of the qualitative interviews. All codes can be seen as answer options in the web-based questionnaire and are marked with their origin (literature or interviews). | Multimedia Appendix 4 |
| 12. Critical appraisal of individual sources of evidence | Given the articles included in the review were used to derive thematic codes to inform the creation of the web-based questionnaire, no risk of bias was expected. The findings were not used as primary data to derive ultimate results but used to inform the conduction of primary data collection through other methods (mixed methods approach with qualitative interviews and a quantitative web-based survey). | - |
| 13. Synthesis of results | All articles included in the review and all snowballed articles were recorded in a tabular overview including title, DOI, authors, journal, year of publication, methodology, geographic scope, research focus and derived insights. Key insights were extracted to inform the creation of the web-based questionnaire. | Multimedia Appendix 6 |
| **Section: Results** |  |  |
| 14. Selection of sources of evidence | After removal of duplicates, the 2 search queries resulted in 429 articles included in the prescreening process. After prescreening through filters and abstract review, 115 articles were assessed for eligibility through full text assessment. After careful eligibility selection, 25 articles were included in the review. The details of the selection process including the reasons for exclusion can be found in Figure 1. Backward and forward snowballing was conducted, leading to 12 identified articles. | Figure 1 |
| 15. Characteristics of sources of evidence | All included and snowballed articles were recorded in a tabular overview including title, DOI, authors, journal, year of publication, methodology, geographic scope, research focus and derived insights. The derived insights attributes indicate which findings were used to inform the creation of the web-based questionnaire. Stated types of EMHP were used to identify the most relevant ones while found facilitators and barriers were used to define the initial codes. | Multimedia Appendix 6 |
| 16. Critical appraisal within sources of evidence | Given the articles included in the review were used to derive thematic codes to inform the creation of the web-based questionnaire, no risk of bias was expected. The findings were not used as primary data to derive ultimate results but used to inform the conduction of primary data collection through other methods. Therefore, potential bias within individual articles were not relevant. | - |
| 17. Results of individual sources of evidence | The derived insights per article used for the derivation of relevant codes for the creation of the web-based questionnaire are presented in Multimedia Appendix 6. The concrete codes derived from the literature can be found in Multimedia Appendix 4. | Multimedia Appendix 4;  Multimedia Appendix 6 |
| 18. Synthesis of results | A tabular overview of the included and snowballed articles informs about the derived insights. | Multimedia Appendix 6 |
| **Section: Discussion** |  |  |
| 19. Summary of evidence | The scoping review revealed 25 relevant articles published between 2018 and 2023 researching different kinds of EMHPs. 12 articles were identified through snowballing. The findings of the articles were used to inform the creation of the web-based questionnaire. Concretely, the articles informed the definition of the possible dimensions of EMHPs, and the identification of relevant facilitators and barriers. Relevant dimensions of EMHPs were, amongst others, medium, interaction form, addressed stage of mental health, and treatment approach. Relevant facilitators and barriers span a variety of factors including cost, company/leadership support, ease of use, data privacy issues, and stigmatization. The findings and derived initial codes for the web-based questionnaire were validated through the qualitative interviews. All codes are used as answer options in the web-based questionnaire. | Multimedia Appendix 4 |
| 20. Limitations | While the “PubMed” database was carefully chosen for the scoping review and represents a reliable source of scientific articles in the medical/health care area, scoping reviews could search several databases to include as many potential articles as possible. By searching only one database, relevant articles might have potentially been missed. However, through snowballing, other relevant articles were considered and included in the review. Furthermore, the results were derived from primary data collected through the web-based survey. | - |
| 21. Conclusions | The findings of the included articles lead to the definition of several EMHP dimensions, including medium, interaction form, and addressed stage of mental health, and to the identification of several facilitators and barriers, which subsequently informed the creation of the web-based questionnaire. As found in existing research and validated through the qualitative interviews, medium, interaction form and addressed stage of mental health were the most relevant dimensions of EMHPs. The review also showed that only limited research is available on several predictors for the use of different types of EMHPs. This research gap was addressed by the present study. | Results |
| **Section: Funding** |  |  |
| 22. Funding | No funding was received for this research. | Declarations |
