# Supplementary material for: Employee Preference and Use of Employee Mental Health Programs: Mixed Methods Study
Source: JMIR Hum Factors. 2025 May 5;12:e65750. doi: 10.2196/65750 (PMC12089874; doi:10.2196/65750)
Supplement: Multimedia Appendix 2 [file humanfactors_v12i1e65750_app2.docx]

**Multimedia Appendix 2. Interview guide for the exploratory qualitative interview study (translated into English given original version was in the German language as interviews were conducted in Germany with German native speakers).**

Context

Before the interviews begin, context on the study is provided, including the objective and approach. Further, a definition of employee mental health program (EMHP) is provided to ensure consistent understanding of what is discussed. The data privacy terms applied to this study were provided in written prior to the interviews.

Types of EMHPs

1. What types of employee health programs in general do you know?
2. What types of employee mental health programs do you know?
3. Which criteria or dimensions are relevant to categorize EMHPs?

Preference for EMHPs

1. If you could create an EMHP completely as to your preference, how would it look like? Which criteria or dimensions would be relevant? Please describe such an EMHP.

Use of EMHPs

1. From your perspective, which factors would increase the probability that employees use an EMHP offered by their employer? Please consider factors that are relevant for you personally, but also factors that might be relevant for other persons.
2. From your perspective, which factors would decrease the probability that employees use an EMHP offered by their employer? Please consider factors that are relevant for you personally, but also factors that might be relevant for other persons.
3. Ease of access: One potential factor impacting use probability is the ease of access. From your perspective, which aspects or conditions would increase ease of access to EMHPs?
4. Ease of access: One potential factor impacting use probability is the ease of access. From your perspective, which aspects or conditions would decrease ease of access to EMHPs?

Relevant mental health disorders in employees

1. Based on your personal perception, which are the most relevant or most prevalent mental health disorders from which employees suffer?

Other aspects regarding EMHPs

1. Which other relevant aspects come to your mind when thinking about EMHPs? Do you have any other thoughts on the topic you would like to share?

Demographic characteristics

1. How old are you?
2. Which gender do you identify with?
3. What is your highest level of formal education?
4. How many employees work at your employer?
5. Which is the industry or sector your employer can best be assigned to?

Termination

The interview is terminated and the interviewer thanks the participant.
