# Supplementary material for: Employee Preference and Use of Employee Mental Health Programs: Mixed Methods Study
Source: JMIR Hum Factors. 2025 May 5;12:e65750. doi: 10.2196/65750 (PMC12089874; doi:10.2196/65750)
Supplement: Multimedia Appendix 3 [file humanfactors_v12i1e65750_app3.docx]

**Multimedia Appendix 3. The 32-item COREQ (Consolidated Criteria for Reporting Qualitative Research) checklist.**

| **Item** | **Guide question** | **Description** | **Item reporting** |
| --- | --- | --- | --- |
| **Domain 1: Research team and reflexivity** | | | |
| *Personal characteristics* | | | |
| 1. Interviewer/facilitator | Which author/s conducted the interview or focus group? | Benedict Sevov conducted the interviews | - |
| 2. Credentials | What were the researcher’s credentials? E.g., PhD, MD | Benedict Sevov holds 1 BA and 2 MSc | - |
| 3. Occupation | What was their occupation at the time of the study? | Benedict Sevov is a PhD candidate at Witten/Herdecke University | Title page |
| 4. Gender | Was the researcher male or female? | Benedict Sevov is male | - |
| 5. Experience and training | What experience or training did the researcher have? | Benedict Sevov has experience in conducting qualitative interviews through interview studies during MSc program and professional experience | - |
| *Relationship with participants* | | | |
| 6. Relationship established | Was a relationship established prior to study commencement? | Participants were recruited from the broader network of all coauthors; therefore, the interviewer knew some of the participants by name and demographic characteristics prior to the study | - |
| 7. Participant knowledge of the interviewer | What did the participants know about the researcher? E.g., personal goals, reasons for doing the research | All participants were briefed on the objective of the conducted research and were provided with the relevant information material (i.e., privacy statement); when asked, the researcher explained that the study was conducted in the context of a PhD program | Methods |
| 8. Interviewer characteristics | What characteristics were reported about the interviewer/ facilitator? E.g., Bias, assumptions, reasons and interests in the research topic | No characteristics on the interviewer were reported as no relevant biases were expected; the interviewer only had scientific interest in the study, there was no economic interest | - |
| **Domain 2: Study design** | | | |
| *Theoretical framework* | | | |
| 9. Methodological orientation and theory | What methodological orientation was stated to underpin the study? E.g., grounded theory, discourse analysis, ethnography, phenomenology, content analysis | Directed content analysis and thematic analysis was applied through 2 iterative coding cycles | Methods |
| *Participant selection* | | | |
| 10. Sampling | How were participants selected? E.g., purposive, convenience, consecutive, snowball | A purposive sampling was applied; participants were recruited from the broader network of all coauthors and selected such that a representative sample of the observed population (employees of employers in Germany) was obtained, distributed across demographic and company characteristics (e.g., age, gender, industry of employer) | Methods |
| 11. Method of approach | How were participants approached? E.g., face-to-face, telephone, mail, email | Participants were contacted via email | - |
| 12. Sample size | How many participants were in the study? | 15 participants were interviewed | Results |
| 13. Non-participation | How many people refused to participate or dropped out? Reasons? | All contacted persons were willing to participate in the interview study, none of them dropped out during the interview process | - |
| *Setting* | | | |
| 14. Setting of data collection | Where was the data collected? E.g., home, clinic, workplace | Data was collected either in personal settings in private residence rooms of the participants or via phone or video calls | Methods |
| 15. Presence on non-participants | Was anyone else present besides the participants and researchers? | No, only Benedict Sevov and the respective participant were present | - |
| 16. Description of sample | What are the important characteristics of the sample? E.g., demographic data, date | The sample was well distributed across relevant defined characteristics, e.g., age, gender, education, number of employees, industry | Multimedia Appendix 7 |
| *Data collection* | | | |
| 17. Interview guide | Were questions, prompts, guides provided by the authors? Was it pilot tested? | Interviews were semistructured based on a prepared interview guide which was tested and iterated by coauthors; results of questions 1. to 3. and 11. to 15. were also used for another research study, but different analysis approaches were applied | Methods; Multimedia Appendix 2 |
| 18. Repeat interviews | Were repeat interviews carried out? If yes, how many? | No repeat interviews were required | - |
| 19. Audio/visual recording | Did the research use audio or visual recording to collect the data? | Interviews, both, face-to-face and via phone or video call, were audio recorded | Methods |
| 20. Field notes | Were field notes made during and/or after the interview or focus group? | No field notes were made, the audio recordings were transcribed after the interviews | - |
| 21. Duration | What was the duration of the interviews or focus group? | Interview durations ranged from 25 to 46 minutes | - |
| 22. Data saturation | Was data saturation discussed? | Data saturation was not discussed with participants | - |
| 23. Transcripts returned | Were transcripts returned to participants for comment and/or correction? | Transcripts were not returned to participants | - |
| **Domain 3: Analysis and findings** | | | |
| *Data analysis* | | | |
| 24. Number of data coders | How many data coders coded the data? | 2 authors (Benedict Sevov, Robin Huettemann) coded and reviewed the data independently | Methods |
| 25. Description of the coding tree | Did authors provide a description of the coding tree? | Directed content analysis and thematic analysis was conducted, the coding approach is described in the methods section | Methods |
| 26. Derivation of themes | Were themes identified in advance or derived from the data? | For both approaches, themes were derived in advance based on literature but also, new themes were derived from the data to complement the themes from the literature | Methods |
| 27. Software | What software, if applicable, was used to manage the data? | MAXQDA was used to manage and analyze the data; Microsoft Word was used to create the transcripts | Methods |
| 28. Participant checking | Did participants provide feedback on the findings? | No | - |
| *Reporting* | | | |
| 29. Quotations presented | Were participant quotations presented to illustrate the themes/findings?  Was each quotation identified? E.g., participant number | Quotations were presented to support the discussion of the findings; each quotation was referenced with the unique factually anonymized ID (number) of the respective participant | Discussion |
| 30. Data and findings consistent | Was there consistency between the data presented and the findings? | Study findings were reported such that they are consistent with the collected data | - |
| 31. Clarity of major themes | Were major themes clearly presented in the findings? | Major themes informed the finalization of the web-based questionnaire and are represented in the findings, e.g., through quotes in the discussion | - |
| 32. Clarity of minor themes | Is there a description of diverse cases or discussion of minor themes? | Not applicable | - |
