# Supplementary material for: Employee Preference and Use of Employee Mental Health Programs: Mixed Methods Study
Source: JMIR Hum Factors. 2025 May 5;12:e65750. doi: 10.2196/65750 (PMC12089874; doi:10.2196/65750)
Supplement: Multimedia Appendix 4 [file humanfactors_v12i1e65750_app4.docx]

**Multimedia Appendix 4. Web-based questionnaire for the quantitative web-based survey (translated into English given original version was in the German language as web-based survey was conducted in Germany; code origin indicated by “[L]” for literature review and “[I]” for qualitative interview study).**

Landing page

Dear Madam or Sir,

Thank you for your interest in this study. This study aims to generate scientific insights about employee mental health programs that are offered by employers.

For this purpose, we invite you to participate in this web-based survey, which will take around 10 to 15 minutes of your time. In this web-based survey we ask you questions about concrete characteristics of employee mental health programs, about personal preference and use with regards to such programs, and about relevant factors impacting the use of such programs.

If you take part in this web-based survey, please take your time to answer each question to the best of your understanding. All questions regarding your employer are referring to your current employer unless stated otherwise.

This web-based survey is designed for participation via desktop devices as well as via mobile devices. However, for a smoother interface, we recommend participating via desktop computer.

A positive ethics vote not raising any ethical concerns regarding this study was received by the Ethics Committee of Witten/Herdecke University (S-12/2023).

Below you find all relevant information regarding data privacy. To participate in this web-based survey, you need to consent with the displayed terms by checking the checkbox.

[Data privacy terms are displayed; a provided link opens up a pop-up window with a PDF file containing the data privacy terms, which can be downloaded]

Employment

1. Are your currently employed by an employer in Germany?
   1. Yes [required to continue]
   2. No
2. Are you employed by your current employer for at least six months?
   1. Yes [required to continue]
   2. No

[Only participants answering “Yes” to both questions are allowed to continue the web-based survey; for others, the web-based survey is terminated]

Demographic characteristics

1. To which age group do you belong?
   1. 29 years or below
   2. 30 to 39 years
   3. 40 to 49 years
   4. 50 to 59 years
   5. 60 years or above
   6. Prefer not to answer
2. Which gender do you identify with?
   1. Woman
   2. Man
   3. Nonbinary
   4. Prefer not to answer
3. What is your gross annual income (before taxes) from your employment?
   1. EUR 0-19,999
   2. EUR 20,000-39,999
   3. EUR 40,000-59,999
   4. EUR 60,000-79,999
   5. EUR 80,000-99,999
   6. EUR min. 100,000
   7. Prefer not to answer
4. What is your highest level of formal education?
   1. None
   2. School degree except high school (“Abitur”)
   3. High school degree (“Abitur”) or equivalent
   4. Professional degree, vocational training, or equivalent
   5. Bachelor’s degree or equivalent
   6. Master’s degree or equivalent
   7. Doctor or PhD degree or equivalent
   8. Prefer not to answer

Digital health care literacy

To what extent do you agree with the following statements?
[Evaluation on a 7-point Likert scale: 1 “Strongly disagree” to 7 “Strongly agree”]

1. I can use applications/programs (like Zoom) on my cell phone, computer, or another electronic device on my own (without asking for help from someone else).
2. I can set up a video chat using my cell phone, computer, or another electronic device on my own (without asking for help from someone else).
3. I can solve or figure out how to solve basic technical issues on my own (without asking for help from someone else).

Personal mental health

Please answer the following questions:

1. How much of the time during the last 4 weeks have you felt downhearted and blue?
   1. None of the time
   2. A little of the time
   3. Some of the time
   4. A good bit of the time
   5. Most of the time
   6. All of the time
   7. Prefer not to answer
2. How much of the time during the last 4 weeks have you felt so down in the dumps that nothing could cheer you up?
   1. None of the time
   2. A little of the time
   3. Some of the time
   4. A good bit of the time
   5. Most of the time
   6. All of the time
   7. Prefer not to answer
3. How much of the time during the last 4 weeks have you been a happy person?
   1. None of the time
   2. A little of the time
   3. Some of the time
   4. A good bit of the time
   5. Most of the time
   6. All of the time
   7. Prefer not to answer
4. Have you ever experienced issues with your mental health in the past?
   1. Yes
   2. No
   3. Prefer not to answer
5. Do you currently or in general practice any activities related to mental health, e.g., psychotherapy, meditation, mindfulness exercises, mobile app on mental health?
   1. Yes
   2. No
   3. Prefer not to answer
6. Have you ever used any employee mental health program provided by your current or former employer to help preventing or treating mental health issues?
   1. Yes
   2. No
   3. Prefer not to answer

Relevance of mental health

Please answer the following question:
[Evaluation on a 7-point Likert scale: 1 “Very irrelevant” to 7 “Very relevant”]

1. From your perspective, how relevant is the topic mental health for society?
2. Based on your personal perception, which are the most relevant or most prevalent mental disorders employees experience?
   [Displayed in randomized order to prevent bias in answer selection]
   1. Depression [L]
   2. Burnout [L]
   3. Anxiety disorders [L]
   4. Insomnia [L]
   5. Eating disorders [L]
   6. Substance abuse (e.g., alcohol addiction, drug addiction) [L]
   7. Paranoia [L]
   8. Post-traumatic stress disorder [L]
   9. Schizophrenia [L]
   10. Obsessive-compulsive disorder [L]
   11. Boreout [I]
   12. Concentration issues [I]
   13. Aggressive behavior [I]
   14. Other [Free text field]

Company- and profession-related characteristics

1. How many employees work at your employer?
   1. 9 or less
   2. 10-49
   3. 50-249
   4. 250-499
   5. 500-999
   6. 1000-9999
   7. 10,000 or more
2. In which industry or sector does your employer operate? Please choose the option that applies best.
   1. Energy and utilities
   2. Materials (raw and basic materials) as well as agriculture, forestry, fishing
   3. Industrial goods and services: manufacturing of goods for industrial/commercial and public sector and associated services as well as transportation and construction
   4. Consumer goods and services as well as retail
   5. Commercial, technical, and scientific services as well as creative services
   6. Health care sector
   7. Finance, insurance, and real estate
   8. Information technology, media, and communication services
   9. Public/governmental institutions including public schools and kindergartens
   10. Association, federation, foundation
   11. Other
3. What type of profession does best describe your role or what you do? Please choose the option that applies best.
   1. State officials and state employed professions including soldiers and administrative employees
   2. Scientists and researchers
   3. Commercial professions and office workers as well as associated professions
   4. Technicians and associated professions
   5. Service professions (e.g., hospitality) and retail workers
   6. Social, teaching and caring professions
   7. Creative and artistic professions
   8. Handcraft and associated professions as well as plant and machine operators
   9. Skilled agricultural, forestry and fishery workers
   10. Other

Company culture

To what extent do you agree with the following statements regarding your current employer?
[Evaluation on a 7-point Likert scale: 1 “Strongly disagree” to 7 “Strongly agree”]

1. In case of errors and issues, the first thing being done is to look for causes.
2. Informing employees has a high value.
3. Managers place great trust in the employees.
4. The employees are involved in decisions.
5. The leadership style is cooperative.
6. Conflicts are addressed openly.
7. The company is characterized by team orientation.
8. The relationship between employees is characterized by cooperation.
9. The employees place great trust in the managers.

Control question

Control questions: Please choose answer option 2 (second option from the left in your browser; second option from the top on your smartphone) on the scale to demonstrate that you are attentive when completing the web-based survey.
[Evaluation on a 7-point Likert scale: 1 “1” to 7 “7”]

1. Please choose option 2

Employee mental health programs – definitions

Please be aware of the following definitions of specific characteristics of employee mental health programs when answering the questions of the following sections of this web-based survey.

Medium

Digital program: any digital technology is used (even if only one component of the program is digital), e.g., mobile app

Analog program: program works completely without digital components, e.g., personal face-to-face session with psychological professional

Interaction form

Self-intervention program: no other person is involved in the use, e.g., use of mobile app with video content

Bilateral intervention program: exactly one other person (interaction partner) is involved in the use, e.g., one-on-one session with psychological professional

Group intervention program: more than one other person is involved in the use, e.g., group sessions or events with peers (e.g., colleagues) with or without professional guidance for the group

Application area

Prevention program: target group has no (acute) mental health issue (objective: sustain mental health)

Treatment program: target group has a (acute) mental health issue (objective: regain mental health)

Rehabilitation program: target group had a (acute) mental health issue (objective: sustain regained mental health and prevent recurrence)

Employee mental health programs – questions (1/4)

Please imagine your current employer offers an employee mental health program. To what extent do you agree with the following statements?
[Evaluation on a 7-point Likert scale: 1 “Strongly disagree” to 7 “Strongly agree”]

Generally, I could imagine using…

1. … a digital self-intervention program
2. … a digital bilateral intervention program (one interaction partner)
3. … a digital group intervention program
4. … an analog self-intervention program
5. … an analog bilateral intervention program (one interaction partner)
6. … an analog group intervention program

Please imagine your current employer offers an employee mental health program. To what extent do you agree with the following statements?
[Evaluation on a 7-point Likert scale: 1 “Strongly disagree” to 7 “Strongly agree”]

Generally, I could imagine using an employee mental health program…

1. … to prevent mental health issues (sustaining mental health)
2. … to treat existing mental health issues (regaining mental health)
3. … to rehabilitate overcome mental health issues (sustaining regained mental health)

Employee mental health programs – questions (2/4)

1. If you could choose one specific employee mental health program that your employer would offer, which of the following programs would best represent your preference or which one would you most probably use?
   1. Digital self-intervention program
   2. Digital bilateral intervention program
   3. Digital group intervention program
   4. Analog self-intervention program
   5. Analog bilateral intervention program
   6. Analog group intervention program

Employee mental health programs – questions (3/4)

1. Facilitators: Which factors would increase the probability that you would use the program you prefer? Please select the most relevant factors, which are important to you personally. [Participants had to choose at least one factor in order to continue with the web-based survey; the maximum number to select was 5 factors]
   [Displayed in randomized order to prevent bias in answer selection]
   1. Fully or partly paid for by employer [L]
   2. (Proven) Effectiveness of program [L]
   3. Fun/enjoyment when using program [L]
   4. High quality/interesting content of program [L]
   5. Easy access to program [L]
   6. Ease of use/appropriate effort required to use program [L]
   7. Reminders to employees to use program/do exercises [L]
   8. Trusted data protection/anonymous use of program [L]
   9. Managers use/recommend program [L]
   10. Colleagues use/recommend program [L]
   11. Supported by company culture/leadership [L]
   12. Personalized content/program [L]
   13. Appropriate time required/flexibility to use program [L]
   14. Program takes place outside of workplace or in secured/separated location [L]
   15. Friends/personal contacts use/recommend program [L]
   16. Mandatory program/intervention [L]
   17. Program functions technically [I]
   18. Awareness for program/topic / good communication [I]
   19. Use together/in group and/or actively [I]
   20. Program can be used during working hours [I]
   21. Recommendation by health care stakeholders, e.g., medical doctor, health insurance [I]
   22. Mental health program in combination with physical health program [I]
   23. Dedicated contact person [I]
   24. Offering/selection of several programs [I]
   25. Opportunity to test program [I]
   26. Continuity in program [I]
   27. (Monetary) Incentivization/gift or voucher cards for use [I]
   28. Other [Free text field]

Employee mental health programs – questions (4/4)

1. Barriers: Which factors would decrease the probability that you would use the program you prefer? Please select the most relevant factors, which are important to you personally. [Participants had to choose at least one factor in order to continue with the web-based survey; the maximum number to select was 5 factors]
   [Displayed in randomized order to prevent bias in answer selection]
   1. Offered program not known [L]
   2. No need for program to support mental health/no mental health issues [L]
   3. Employee must pay or partly pay for it [L]
   4. No or low effectiveness of program [L]
   5. Symptoms of mental health issues which decrease motivation [L]
   6. Difficult/complicated access to program [L]
   7. High effort required/complicated to use program [L]
   8. Too much time required/not enough time/increasing pressure or load [L]
   9. Low quality of content of program [L]
   10. Company culture/management not supportive [L]
   11. Colleagues not supportive, socially not accepted [L]
   12. Stigmatization/negative bias towards topic [L]
   13. Lack of technical/digital skills [L]
   14. Use of other resources in private life [L]
   15. Program not tailored to individual requirements/desires [L]
   16. Data protection/privacy concerns/program cannot be used anonymously [L]
   17. Technical malfunctions [L]
   18. Expectation/worry of negative consequences through employer [L]
   19. No personal connection with coach/guide/leader of program [L]
   20. Feeling forced to use program [I]
   21. Program to be used outside of working hours [I]
   22. No continuous program / only onetime event [I]
   23. Other [Free text field]

Availability of employee mental health program at employer

1. Does your current employer offer any employee mental health program to its employees or did so in the last 12 months, e.g., mobile app on mental health, sessions with psychiatrist/coach?
   1. Yes
   2. No
   3. Do not know

[Only participants answering “Yes” are allowed to continue the web-based survey; for others, the web-based survey is terminated]

1. Which of the following characteristics best describe the employee mental health program offered at your employer? If more than one program is offered, please refer to the program you prefer or know most about.
   1. Digital
   2. Analog
   3. Do not know
2. Which of the following characteristics best describe the employee mental health program offered at your employer? If more than one program is offered, please refer to the program you prefer or know most about.
   1. Self-intervention
   2. Bilateral intervention (one interaction partner)
   3. Group intervention
3. Which of the following characteristics best describe the employee mental health program offered at your employer? If more than one program is offered, please refer to the program you prefer or know most about.
   1. Focus on prevention
   2. Focus on treatment
   3. Focus on rehabilitation
4. Do you use at least one employee mental health program offered by your current employer or did you in the last 12 months?
   1. Yes
   2. No
   3. Prefer not to answer

[Only participants answering “Yes” are able to see the following question; for others, the web-based survey is terminated]

To what extent do you agree with the following statements regarding the employee mental health program that you are using or used in the last 12 months?
[Evaluation on a 7-point Likert scale: 1 “Strongly disagree” to 7 “Strongly agree”]

1. All things considered, I am very satisfied with the program.
2. Overall, my interaction with the program is very satisfying.

Final prompt

You successfully completed the web-based survey. Thank you very much for your time and participation. You can now close your browser window.
