# Supplementary material for: Employee Preference and Use of Employee Mental Health Programs: Mixed Methods Study
Source: JMIR Hum Factors. 2025 May 5;12:e65750. doi: 10.2196/65750 (PMC12089874; doi:10.2196/65750)
Supplement: Multimedia Appendix 5 [file humanfactors_v12i1e65750_app5.docx]

**Multimedia Appendix 5. The 30-item CHERRIES (Checklist for Reporting Results of Internet E-Surveys) checklist.**

| **Item** | **Description** |
| --- | --- |
| **Category: Design** | |
| 1. Describe survey design | The target population were persons employed by an employer in Germany for a minimum of 6months (selection criteria described in Methods part) |
| **Category: IRB (Institutional Review Board) approval and informed consent process** | |
| 2. IRB approval | The Ethics Committee of Witten/Herdecke University raised no ethical concerns with regard to this research project (S-12/2023). |
| 3. Informed consent | Participants were provided with information material and the privacy statement; the provided information disclosed the name of the responsible researchers and institution, explained the purpose of the study, disclosed the estimated duration of survey participation as well as all details regarding data protection including data collected and stored; participants provided voluntary, informed, and written consent to participate in the study and have the results published in a peer-reviewed article |
| 4. Data protection | Personal data (demographic characteristics, e.g., age, gender) including special personal data (health data, e.g., mental health status) were collected based on the General Data Protection Regulation; the data collection approach was approved by the data protection officer of Witten/Herdecke University; data collected were anonymized using k≥5 anonymity technique; demographic characteristics such as age were collected in predefined intervals, not as exact values (e.g., age intervals, not year of age); at no time the researchers were able to trace back answers to individual participants |
| **Category: Development and pretesting** | |
| 5. Development and testing | The questionnaire was developed based on a scoping review; the drafted questionnaire was validated and adjusted based on semistructured qualitative interviews; the final questionnaire was tested (content-wise and technically) by all coauthors and selected testers from the target population; a first sample of 300 participants was analyzed before completing the data collection to obtain the targeted sample size of >1000 participants |
| **Category: Recruitment process and description of the sample having access to the questionnaire** | |
| 6. Open survey vs. closed survey | The data was collected through the German research panel provider Splendid Research GmbH that invited registered panel participants from across Germany; the authors remunerated the research panel provider for its services (participant recruitment and sampling approach described in Methods part) |
| 7. Contact mode | Participants were contacted and recruited via email by the German research panel provider Splendid Research GmbH that conducted the data collection (participant recruitment and sampling approach described in Methods part) |
| 8. Advertising the survey | The survey was advertised by the German research panel provider Splendid Research GmbH that contacted registered panel participants via email to invite them to participate in the survey |
| **Category: Survey administration** | |
| 9. Web/Email | The survey was a web survey hosted on a website through the web-based service tool LimeSurvey; data was directly recorded by LimeSurvey when participants entered their answers |
| 10. Context | The survey website provided relevant information (information material and privacy statement) on the study and collected consent of the participant prior to survey participation; persons not employed in Germany for a minimum of 6 months were excluded after the respective screening questions (context of survey administration described in Methods part) |
| 11. Mandatory/voluntary | The survey was based on voluntary participation |
| 12. Incentives | The participants received a monetary compensation from the German research panel provider Splendid Research GmbH that conducted the data collection |
| 13. Time/date | The survey was conducted in April and May 2023 (time of survey conduction stated in Methods part) |
| 14. Randomization of items or questionnaires | Answer items of the questions on the most relevant mental health indications and on relevant factors for the use of EMHPs (facilitators and barriers) were randomized to prevent biases in answers |
| 15. Adaptive questioning | Participants were screened through 2 screening questions at the beginning of the survey to ensure that only people employed by an employer in Germany for a minimum of 6 months participated in the study, for participants not meeting these criteria the survey was immediately terminated; participants whose employer offers at least one EMHP were asked specific questions about the EMHP, these questions were only displayed to the respective participants; participants who used an EMHP offered by their employer were asked about their satisfaction with the program, this question was only displayed to these respective participants |
| 16. Number of items | The survey consisted of 49 items (including screening questions and questions on demographic characteristics) of which 43 were displayed to all participants (included after the screening questions) |
| 17. Number of screens (pages) | The web-based questionnaire was presented on 13 screens (web pages) displaying question items (not considering intro page with information material and privacy statement) |
| 18. Completeness check | Automated completeness checks were technically integrated into the web-based survey such that participants could only move to the next screen or question when all answers were given; option “Prefer not to answer” was given for selected questions |
| 19. Review step | Participants were able to use a “Back” button to see or change their previous answers |
| **Category: Response rates** | |
| 20. Unique site visitor | Unique survey participation was ensured by the research panel provider |
| 21. View rate (ratio unique site visitors/unique survey visitors) | A view rate was not assessed |
| 22. Participation rate (ratio unique survey page visitors/agreed to participate) | A participation rate was not assessed |
| 23. Completion rate (ratio agreed to participate/finished survey) | A completion rate was not assessed |
| **Category: Preventing multiple entries from the same individual** | |
| 24. Cookies used | Cookies were not used to ensure a maximum level of data privacy |
| 25. IP check | IP addresses were not collected to ensure a maximum level of data privacy |
| 26. Log file analysis | A log file analysis was not included in the study |
| 27. Registration | Participants (panel participants of the research panel provider) were not able to access the survey a second time after completion according to the research panel provider |
| **Category: Analysis** | |
| 28. Handling of incomplete questionnaires | Only completed surveys were analyzed, incomplete surveys were not considered (data cleaning process depicted in Figure 2) |
| 29. Questionnaires submitted with an atypical timestamp | Completed surveys with completion time below the threshold of the fastest 10% of the first 1000 participants who completed the survey were not considered for analysis (data cleaning regarding completion time threshold described in Figure 2) |
| 30. Statistical correction | The Greenhouse-Geisser correction was applied when the assumption of sphericity was violated; the Bonferroni correction was applied for multiple comparisons |
