# Supplementary material for: Employee Preference and Use of Employee Mental Health Programs: Mixed Methods Study
Source: JMIR Hum Factors. 2025 May 5;12:e65750. doi: 10.2196/65750 (PMC12089874; doi:10.2196/65750)
Supplement: Multimedia Appendix 6 [file humanfactors_v12i1e65750_app6.docx]

**Multimedia Appendix 6. Overview of the literature identified through scoping review and snowballing.**

| **Item** | **Title** | **DOI** | **Authors** | **Journal** | **Year of publication** | **Methodology** | **Geographic scope** | **Research focus** | **Derived insights** |  |
| --- | --- | --- | --- | --- | --- | --- | --- | --- | --- | --- |
| *Scoping review* | | | | | | | | | | |
| 1 | The Effectiveness of Digital Interventions for Psychological Well-Being in the Workplace: A Systematic Review Protocol | 10.3390/ijerph17010255 | Armaou et al. | International Journal of Environmental Research and Public Health | 2019 | Systematic literature review | Global | Effectiveness of EMHPs | Types of EMHPs |  |
| 2 | A Global Perspective on Promoting Workplace Mental Health and the Role of Employee Assistance Programs | 10.1177/0890117119838101c | Attridge | American Journal of Health Promotion | 2019 | Status quo assessment | Global | Meaning of EMHPs on promoting mental health at the workplace | Types of EMHPs |  |
| 3 | Interventions for common mental disorders in the occupational health service: a systematic review with a narrative synthesis | 10.1007/s00420-020-01535-4 | Axén et al. | International Archives of Occupational and Environmental Health | 2020 | Systematic literature review | Global | Effectiveness of EMHPs | Types of EMHPs |  |
| 4 | Professional training in mental health self-care for nurses starting work in hospital departments | 10.3233/WOR-203311 | Bernburg et al. | Work | 2020 | Randomized controlled trial | Germany | Effectiveness of EMHPs on health care workers | Types of EMHPs; focus on specific groups |  |
| 5 | Contextualising the Effectiveness of an Employee Assistance Program Intervention on Psychological Health: The Role of Corporate Climate | 10.3390/ijerph19095067 | Bouzikos et al. | International Journal of Environmental Research and Public Health | 2022 | Web-based survey | Australia, New Zealand | Effectiveness of EMHPs | Types of EMHPs; relevant factor company culture |  |
| 6 | Economic analyses of mental health and substance use interventions in the workplace: a systematic literature review and narrative synthesis | 10.1016/S2215-0366(20)30145-0 | de Oliveira et al. | The Lancet Psychiatry | 2020 | Systematic literature review | Global | Economic analyses of EMHPs | Types of EMHPs |  |
| 7 | With a little help from my boss: The impact of workplace mental health training on leader behaviors and employee resource utilization | 10.1037/ocp0000126 | Dimoff & Kelloway | Journal of Occupational Health Psychology | 2019 | Randomized controlled trial | Canada | Impact of leadership on employees' use of EMHPs | Facilitators for EMHP use |  |
| 8 | Effects of the Anti-stigma Workplace Intervention "Working Mind" in a Canadian Health-Care Setting: A Cluster-Randomized Trial of Immediate Versus Delayed Implementation | 10.1177/0706743720961738 | Dobson et al. | Canadian Journal of Psychiatry | 2021 | Cluster randomized trial | Canada | Effectiveness of EMHPs | Types of EMHPs; relevant factor stigmatiza-tion |  |
| 9 | Workplace Support for Mental Health Workers Who Are Parents: A Feasibility Study | 10.3389/fpsyg.2022.8540652 | Dunn et al. | Frontiers in Psychology | 2022 | Uncontrolled evaluation through self-reporting measures | UK | Effectiveness of EMHPs | Types of EMHPs; focus on specific groups |  |
| 10 | Psychotherapeutic Consultation Services in the Workplace: A Longitudinal Analysis of Treatments and Sick Leave Using Health Insurance Data | 10.3389/fpsyt.2022.838823 | Gantner et al. | Frontiers in Psychiatry | 2022 | Naturalistic longitudinal design analyzing health insurance data | Germany | Effectiveness of EMHPs; characteristics of users | Types of EMHPs |  |
| 11 | Mental Health in the Workplace: A Call to Action Proceedings From the Mental Health in the Workplace-Public Health Summit | 10.1097/JOM.0000000000001271 | Goetzel et al. | Journal of Occupational and Environmental Medicine | 2018 | Advisory council with experts around occupational health | USA | Workplace health promotion through effective programs | Types of EMHPs |  |
| 12 | Workplace-Based Organizational Interventions Promoting Mental Health and Happiness among Healthcare Workers: A Realist Review | 10.3390/ijerph16224396 | Gray et al. | International Journal of Environmental Research and Public Health | 2019 | Systematic literature review | Global | Effectiveness of EMHPs on health care workers | Types of EMHPs |  |
| 13 | Testing the Pragmatic Effectiveness of a Consumer-Based Mindfulness Mobile App in the Workplace: Randomized Controlled Trial | 10.2196/38903 | Huberty et al. | JMIR mHealth and uHealth | 2022 | Randomized controlled trial | USA | Effectiveness of EMHPs | Types of EMHPs |  |
| 14 | The Impact of Personalized Human Support on Engagement With Behavioral Intervention Technologies for Employee Mental Health: An Exploratory Retrospective Study | 10.3389/fdgth.2022.846375 | Jesuthasan et al. | Frontiers in Digital Health | 2022 | Exploratory retrospective design | Malaysia | Factors for engagement with EMHPs | Facilitators for EMHP use |  |
| 15 | Effects of gratitude intervention on mental health and well-being among workers: A systematic review | 10.1002/1348-9585.12290 | Komase et al. | Journal of Occupational Health | 2021 | Systematic literature review | Global | Effectiveness of gratitude EMHP | Types of EMHPs |  |
| 16 | A multi-faceted community intervention is associated with knowledge and standards of workplace mental health: the Superior Mental Wellness @ Work study | 10.1186/s12889-019-6976-x | Kristman et al. | BMC Public Health | 2019 | Quasi-experimental design | Canada | Program effectiveness on mental health awareness and knowledge | Types of EMHPs |  |
| 17 | Efficacy of a Workplace Intervention Program With Web-Based Online and Offline Modalities for Improving Workers' Mental Health | 10.3389/fpsyt.2022.888157 | Lam et al. | Frontiers in Psychiatry | 2022 | Randomized controlled trial | Australia | Effectiveness of EMHPs | Types of EMHPs |  |
| 18 | Mental health promotion for junior physicians working in emergency medicine: evaluation of a pilot study | 10.1097/MEJ.0000000000000434 | Mache et al. | European Journal of Emergency Medicine | 2018 | Randomized controlled trial | Germany | Effectiveness of EMHPs on health care workers | Types of EMHPs; focus on specific groups |  |
| 19 | Exploring men's use of mental health support offered by an Australian Employee Assistance Program (EAP): perspectives from a focus-group study with males working in blue- and white-collar industries | 10.1186/s13033-021-00489-5 | Matthews et al. | International Journal of Mental Health Systems | 2021 | Qualitative focus groups | Australia | Factors for EMHP use for male employees | Barriers for EMHP use |  |
| 20 | The effectiveness of workplace health promotion program in reducing work-related depression, anxiety and stress among manufacturing workers in Malaysia: mixed-model intervention | 10.1007/s00420-022-01836-w | Mohamed et al. | International Archives of Occupational and Environmental Health | 2022 | Randomized controlled trial | Malaysia | Effectiveness of EMHPs on manufacturing workers | Types of EMHPs; focus on specific groups |  |
| 21 | Effects of a work-related stress model based mental health promotion program on job stress, stress reactions and coping profiles of women workers: a control groups study | 10.1186/s12889-020-09769-0 | Ornek & Esin | BMC Public Health | 2020 | Pretest–posttest nonequivalent control group design | n/a | Effectiveness of EMHPs on female workers | Types of EMHPs; focus on specific groups |  |
| 22 | Workplace Mental Health Interventions in India: A Rapid Systematic Scoping Review | 10.3389/fpubh.2022.800880 | Pandya et al. | Frontiers in Public Health | 2022 | Systematic literature review | India | EMHP landscape in India | Types of EMHPs |  |
| 23 | Beyond the Lab: Empirically Supported Treatments in the Real World | 10.3389/fpsyg.2020.01969 | Schneider et al. | Frontiers in Psychology | 2020 | Retrospective design | USA | Effectiveness of EMHPs | Types of EMHPs |  |
| 24 | Systematic review of universal and targeted workplace interventions for depression | 10.1136/oemed-2017-104532 | Wan Mohd Yunus et al. | Occupational and Environmental Medicine | 2018 | Systematic literature review | Global | Landscape and effectiveness of EMHPs | Types of EMHPs |  |
| 25 | Effect of EAP Psychological Intervention on Improving the Mental Health of Medical Workers Under the Novel Coronavirus Epidemic in China | 10.3389/fpubh.2021.6491572 | Xu et al. | Frontiers in Public Health | 2021 | Interventional clinical observation and psychological evaluation through survey | China | Effectiveness of EMHPs on health care workers | Types of EMHPs; focus on specific groups |  |
| *Snowballing* | | | | | | | | | | |
| 1 | Improving Employee Well-Being and Effectiveness: Systematic Review and Meta-Analysis of Web-Based Psychological Interventions Delivered in the Workplace | 10.2196/jmir.7583 | Carolan et al. | Journal of Medical Internet Research | 2017 | Systematic literature review | Global | Effectiveness of EMHPs | Types of EMHPs |  |
| 2 | Employees' Perspectives on the Facilitators and Barriers to Engaging With Digital Mental Health Interventions in the Workplace: Qualitative Study | 10.2196/mental.9146 | Carolan & de Visser | JMIR Mental Health | 2018 | Qualitative interview study | UK | Factors for EMHP use | Facilitators and barriers for EMHP use |  |
| 3 | Predictors of Psychological Distress and Mental Health Resource Utilization among Employees in Malaysia | 10.3390/ijerph18010314 | Chan et al. | International Journal of Environmental Research and Public Health | 2021 | Cross-sectional questionnaire study based on existing database | Malaysia | Factors for EMHP use | Use of EMHPs by specific groups |  |
| 4 | Barriers to Mental Health Service Use Among Workers With Depression and Work Productivity | 10.1097/JOM.000000000000047 | Dewa & Hoch | Journal of Occupational and Environmental Medicine | 2015 | Quantitative model based on survey data | Canada | Factors for EMHP use; economic effect of removing barriers | Barriers for EMHP use |  |
| 5 | Evaluating the effectiveness of employee assistance programmes: a systematic review | 10.1080/1359432X.2017.1374245 | Joseph et al. | European Journal of Work and Organizational Psychology | 2017 | Systematic literature review | Global | Effectiveness of EMHPs | Types of EMHPs |  |
| 6 | The Effect of Employee Assistance Services on Reductions in Employee Absenteeism | 10.1007/s10869-017-9518-5 | Nunes et al. | Journal of Business and Psychology | 2017 | Quasi-experimental design | USA | Effectiveness of EMHPs | Types of EMHPs |  |
| 7 | Worker Preferences for a Mental Health App Within Male-Dominated Industries: Participatory Study | 10.2196/mental.8999 | Peters et al. | JMIR Mental Health | 2018 | Exploratory qualitative  study | Australia | Preference of employees for different types of mental health programs by gender | Types of EMHPs; focus on specific groups |  |
| 8 | Effectiveness of occupational e-mental health interventions: a systematic review and meta-analysis of randomized controlled trials | 10.5271/sjweh.3839 | Phillips et al. | Scandinavian Journal of Work, Environment & Health | 2019 | Systematic literature review | Global | Effectiveness of EMHPs | Types of EMHPs |  |
| 9 | Acceptability of Web-Based Mental Health Interventions in the Workplace: Systematic Review | 10.2196/34655 | Scheutzow et al. | JMIR Mental Health | 2022 | Systematic literature review | Global | Acceptability and use factors of employees regarding EMHPs | Types of EMHPs |  |
| 10 | Health-Related Internet Usage and Design Feature Preference for E-Mental Health Programs Among Men and Women | 10.2196/11224 | Smail-Crevier et al. | Journal of Medical Internet Research | 2019 | Interview study | Canada | Preference for different types of EMHPs by gender | Types of EMHPs; focus on specific groups |  |
| 11 | Effectiveness of eHealth interventions for reducing mental health conditions in employees: A systematic review and meta-analysis | 10.1371/journal.pone.0189904 | Stratton et al. | PLOS One | 2017 | Participatory design approach | Australia | Preference for different types of EMHPs | Types of EMHPs |  |
| 12 | Support for depression in the workplace: Perspectives  of employees, managers, and OHS personnel | 10.1007/s41542-021-00090-9 | van Eerd et al. | Occupational Health Science | 2021 | Web-based survey and interviews/ focus groups | Canada | Preference and factors for use of mental health resources at the workplace incl. barriers | Barriers for EMHP use; relevant factor company culture |  |

**References**

1. Armaou M, Konstantinidis S, Blake H. The Effectiveness of Digital Interventions for Psychological Well-Being in the Workplace: A Systematic Review Protocol. Int J Environ Res Public Health 2019;17(1):255. PMID:31905882
2. Attridge M. A Global Perspective on Promoting Workplace Mental Health and the Role of Employee Assistance Programs. Am J Health Promot 2019;33(4):622-629. PMID:31006254
3. Axén I, Björk Brämberg E, Vaez M, Lundin A, Bergström G. Interventions for common mental disorders in the occupational health service: a systematic review with a narrative synthesis. Int Arch Occup Environ Health 2020;93(7):823-838. PMID:32246230
4. Bernburg M, Groneberg D, Mache S. Professional training in mental health self-care for nurses starting work in hospital departments. Work 2020;67(3):583-590. PMID:33185622
5. Bouzikos S, Afsharian A, Dollard M, Brecht O. Contextualising the Effectiveness of an Employee Assistance Program Intervention on Psychological Health: The Role of Corporate Climate. Int J Environ Res Public Health 2022;19(9):5067. PMID:35564466
6. de Oliveira C, Cho E, Kavelaars R, Jamieson M, Bao B, Rehm J. Economic analyses of mental health and substance use interventions in the workplace: a systematic literature review and narrative synthesis. Lancet Psychiatry 2020;7(10):893-910. PMID:32949521
7. Dimoff JK, Kelloway EK. With a little help from my boss: The impact of workplace mental health training on leader behaviors and employee resource utilization. J Occup Health Psychol 2019;24(1):4-19. PMID:29939045
8. Dobson KS, Markova V, Wen A, Smith LM. Effects of the Anti-stigma Workplace Intervention “Working Mind” in a Canadian Health-Care Setting: A Cluster-Randomized Trial of Immediate Versus Delayed Implementation. Can J Psychiatry 2021;66(5):495-502. PMID:32960651
9. Dunn A, Dixon C, Thomson A, Cartwright-Hatton S. Workplace Support for Mental Health Workers Who Are Parents: A Feasibility Study. Front Psychol 2022;13:854065. PMID:35814147
10. Gantner M, Jarzcok MN, Schneider J, Brandner S, Gündel H, Wietersheim J von. Psychotherapeutic Consultation Services in the Workplace: A Longitudinal Analysis of Treatments and Sick Leave Using Health Insurance Data. Front. Psychiatry 2022;13:838823. PMID:35401269
11. Goetzel RZ, Roemer EC, Holingue C, Fallin MD, McCleary K, Eaton W, Agnew J, Azocar F, Ballard D, Bartlett J, Braga M, Conway H, Crighton KA, Frank R, Jinnett K, Keller-Greene D, Rauch SM, Safeer R, Saporito D, Schill A, Shern D, Strecher V, Wald P, Wang P, Mattingly CR. Mental Health in the Workplace: A Call to Action Proceedings From the Mental Health in the Workplace-Public Health Summit. J Occup Environ Med 2018;60(4):322-330. PMID:29280775
12. Gray P, Senabe S, Naicker N, Kgalamono S, Yassi A, Spiegel JM. Workplace-Based Organizational Interventions Promoting Mental Health and Happiness among Healthcare Workers: A Realist Review. Int J Environ Res Public Health 2019;16(22):4396. PMID:31717906
13. Huberty JL, Espel-Huynh HM, Neher TL, Puzia ME. Testing the Pragmatic Effectiveness of a Consumer-Based Mindfulness Mobile App in the Workplace: Randomized Controlled Trial. JMIR Mhealth Uhealth 2022;10(9):e38903. PMID:36169991
14. Jesuthasan J, Low M, Ong T. The Impact of Personalized Human Support on Engagement With Behavioral Intervention Technologies for Employee Mental Health: An Exploratory Retrospective Study. Front Digit Health 2022;4:846375. PMID:35574254
15. Komase Y, Watanabe K, Hori D, Nozawa K, Hidaka Y, Iida M, Imamura K, Kawakami N. Effects of gratitude intervention on mental health and well-being among workers: A systematic review. J Occup Health 2021;63(1):e12290. PMID:34762326
16. Kristman VL, Lowey J, Fraser L, Armstrong S, Sawula S. A multi-faceted community intervention is associated with knowledge and standards of workplace mental health: the Superior Mental Wellness @ Work study. BMC Public Health 2019;19(1):638. PMID:31126273
17. Lam LT, Lam MK, Reddy P, Wong P. Efficacy of a Workplace Intervention Program With Web-Based Online and Offline Modalities for Improving Workers’ Mental Health. Front. Psychiatry 2022;13:888157. PMID:35711597
18. Mache S, Bernburg M, Baresi L, Groneberg D. Mental health promotion for junior physicians working in emergency medicine: evaluation of a pilot study. Eur J Emerg Med 2018;25(3):191-198. PMID:27879536
19. Matthews LR, Gerald J, Jessup GM. Exploring men’s use of mental health support offered by an Australian Employee Assistance Program (EAP): perspectives from a focus-group study with males working in blue- and white-collar industries. Int J Ment Health Syst 2021;15:68. PMID:34348756
20. Mohamed AF, Isahak M, Awg Isa MZ, Nordin R. The effectiveness of workplace health promotion program in reducing work-related depression, anxiety and stress among manufacturing workers in Malaysia: mixed-model intervention. Int Arch Occup Environ Health 2022;95(5):1113-1127. PMID:35091853
21. Ornek OK, Esin MN. Effects of a work-related stress model based mental health promotion program on job stress, stress reactions and coping profiles of women workers: a control groups study. BMC Public Health 2020;20:1658. PMID:33148247
22. Pandya A, Khanal N, Upadhyaya M. Workplace Mental Health Interventions in India: A Rapid Systematic Scoping Review. Front Public Health 2022;10:800880. PMID:35592077
23. Schneider RA, Grasso JR, Chen SY, Chen C, Reilly ED, Kocher B. Beyond the Lab: Empirically Supported Treatments in the Real World. Front Psychol 2020;11:1969. PMID:32849153
24. Wan Mohd Yunus WMA, Musiat P, Brown JSL. Systematic review of universal and targeted workplace interventions for depression. Occup Environ Med 2018;75(1):66-75. PMID:29074553
25. Xu J, Liu X, Xiao Y, Fang X, Cheng Y, Zhang J. Effect of EAP Psychological Intervention on Improving the Mental Health of Medical Workers Under the Novel Coronavirus Epidemic in China. Front Public Health 2021;9:649157. PMID:34395355
26. Carolan S, Harris PR, Cavanagh K. Improving Employee Well-Being and Effectiveness: Systematic Review and Meta-Analysis of Web-Based Psychological Interventions Delivered in the Workplace. J Med Internet Res 2017;19(7):e271. PMID:28747293
27. Carolan S, de Visser RO. Employees’ Perspectives on the Facilitators and Barriers to Engaging With Digital Mental Health Interventions in the Workplace: Qualitative Study. JMIR Ment Health 2018;5(1):e8. PMID:29351900
28. Chan CMH, Ng SL, In S, Wee LH, Siau CS. Predictors of Psychological Distress and Mental Health Resource Utilization among Employees in Malaysia. Int J Environ Res Public Health 2021;18(1):314. PMID:33406714
29. Dewa CS, Hoch JS. Barriers to Mental Health Service Use Among Workers With Depression and Work Productivity. J Occup Environ Med 2015;57(7):726-731. PMID:26147540
30. Joseph B, Walker A, Fuller-Tyszkiewicz M. Evaluating the effectiveness of employee assistance programmes: a systematic review. Eur J Work Organ Psychol 2017;27(1):1-15. doi:10.1080/1359432X.2017.1374245
31. Nunes AP, Richmond MK, Pampel FC, Wood RC. The Effect of Employee Assistance Services on Reductions in Employee Absenteeism. J Bus Psychol 2017;33(6):699-709. doi:10.1007/s10869-017-9518-5
32. Peters D, Deady M, Glozier N, Harvey S, Calvo RA. Worker Preferences for a Mental Health App Within Male-Dominated Industries: Participatory Study. JMIR Ment Health 2018;5(2):e30. PMID:29695371
33. Phillips EA, Gordeev VS, Schreyögg J. Effectiveness of occupational e-mental health interventions: a systematic review and meta-analysis of randomized controlled trials. Scand J Work Environ Health 2019;45(6):560-576. PMID:31184758
34. Scheutzow J, Attoe C, Harwood J. Acceptability of Web-Based Mental Health Interventions in the Workplace: Systematic Review. JMIR Ment Health 2022;9(5):e34655. PMID:35544305
35. Smail-Crevier R, Powers G, Noel C, Wang J. Health-Related Internet Usage and Design Feature Preference for E-Mental Health Programs Among Men and Women. J Med Internet Res 2019;21(3):e11224. PMID:30882361
36. Stratton E, Lampit A, Choi I, Calvo RA, Harvey SB, Glozier N. Effectiveness of eHealth interventions for reducing mental health conditions in employees: A systematic review and meta-analysis. PLoS One 2017;12(12):e0189904. PMID:29267334
37. van Eerd D, Cullen K, Irvin E, Le Pouésard M, Gignac M. Support for depression in the workplace: Perspectives of employees, managers, and OHS personnel. Occup Health Sci 2021;5:307-343. doi:10.1007/s41542-021-00090-9
