# Supplementary material for: Employee Preference and Use of Employee Mental Health Programs: Mixed Methods Study
Source: JMIR Hum Factors. 2025 May 5;12:e65750. doi: 10.2196/65750 (PMC12089874; doi:10.2196/65750)
Supplement: Multimedia Appendix 7 [file humanfactors_v12i1e65750_app7.docx]

**Multimedia Appendix 7. Demographic and company-related characteristics of participants in the interview study (N=15).**

| **Variable** | **n** | **%** |
| --- | --- | --- |
| Age (y) |  |  |
| ≤29 | 3 | 20.0 |
| 30-39 | 3 | 20.0 |
| 40-49 | 3 | 20.0 |
| 50-59 | 3 | 20.0 |
| ≥60 | 3 | 20.0 |
| Gender |  |  |
| Woman | 8 | 53.3 |
| Man | 7 | 46.3 |
| Nonbinary | 0 | 0.0 |
| Education |  |  |
| None | 0 | 0.0 |
| School degree except high school (“Abitur”) | 0 | 0.0 |
| High school degree (“Abitur”) or equivalent | 0 | 0.0 |
| Professional degree, vocational training, or equivalent | 4 | 26.7 |
| Bachelor’s degree or equivalent | 2 | 13.3 |
| Master’s degree or equivalent | 8 | 53.3 |
| Doctor or PhD degree or equivalent | 1 | 6.7 |
| Number of employees |  |  |
| ≤9 | 1 | 6.7 |
| 10-49 | 2 | 13.3 |
| 50-249 | 2 | 13.3 |
| 250-499 | 1 | 6.7 |
| 500-999 | 2 | 13.3 |
| 1000-9999 | 2 | 13.3 |
| ≥10,000 | 5 | 33.3 |
| Industry |  |  |
| Energy and utilities | 1 | 6.7 |
| Raw materials and natural products | 0 | 0.0 |
| Industrial goods and services | 4 | 26.7 |
| Consumer goods and services | 2 | 13.3 |
| Commercial, technical, and scientific services as well as creative services | 1 | 6.7 |
| Health care | 2 | 13.3 |
| Financials and real estate | 1 | 6.7 |
| IT and communications | 1 | 6.7 |
| Public and governmental | 2 | 13.3 |
| Association, federation, and foundation | 1 | 6.7 |
| Other | 0 | 0.0 |
