# Supplementary material for: Employee Preference and Use of Employee Mental Health Programs: Mixed Methods Study
Source: JMIR Hum Factors. 2025 May 5;12:e65750. doi: 10.2196/65750 (PMC12089874; doi:10.2196/65750)
Supplement: Multimedia Appendix 8 [file humanfactors_v12i1e65750_app8.docx]

**Multimedia Appendix 8. Reliability and factor analyses of the used constructs.**

| **Construct (measure)** | **Items** | **Factor Loadings^a^** | **Cronbach α** |
| --- | --- | --- | --- |
| **Company culture** based on “Kurzskala zur Erfassung der Unternehmenskultur” (corporate culture scale, | *To what extent do you agree with the following statements regarding your current employer?*  *Answers on a 7-point Likert scale, 1 = Strongly disagree and 7 = Strongly agree* |  |  |
| short form), Jöns, 2005 | In case of errors and issues, the first thing being done is to look for causes. | 0.71 | .94 |
|  | Informing employees has a high value. | 0.84 |  |
|  | Managers place great trust in the employees. | 0.84 |  |
|  | The employees are involved in decisions. | 0.80 |  |
|  | The leadership style is cooperative. | 0.87 |  |
|  | Conflicts are addressed openly. | 0.84 |  |
|  | The company is characterized by team orientation. | 0.87 |  |
|  | The relationship between employees is characterized by cooperation. | 0.80 |  |
|  | The employees place great trust in the managers. | 0.85 |  |
| **Digital health care literacy** based on Digital Health Care Literacy Scale (DHLS), | *To what extent do you agree with the following statements?*  *Answers on a 7-point Likert scale, 1 = Strongly disagree and 7 = Strongly agree* |  |  |
| Nelson et al., 2022 | I can use applications/programs (like Zoom) on my cell phone, computer, or another electronic device on my own (without asking for help from someone else). | 0.89 | .84 |
|  | I can set up a video chat using my cell phone, computer, or another electronic device on my own (without asking for help from someone else). | 0.88 |  |
|  | I can solve or figure out how to solve basic technical issues on my own (without asking for help from someone else). | 0.85 |  |
| **Mental health status** based on 3-item Mental Health Inventory (MHI-3), | *Please answer the following questions.*  *Answers on a 6-point Likert scale, 1 = Never and 7 = Always; option to choose ‘Prefer not to answer’* |  |  |
| Yamazaki et al., 2005 | How much of the time during the last 4 weeks have you felt downhearted and blue?^b^ | 0.91 | .76 |
|  | How much of the time during the last 4 weeks have you felt so down in the dumps that nothing could cheer you up?^b^ | 0.89 |  |
|  | How much of the time during the last 4 weeks have you been a happy person? | 0.65 |  |
| **Satisfaction** based on System Satisfaction construct, Wixom & Todd, 2005 | *To what extent do you agree with the following statements regarding the used program?*  *Answers on a 7-point Likert scale, 1 = Strongly disagree and 7 = Strongly agree* |  |  |
|  | All things considered, I am very satisfied with the program. | 0.87 | .68 |
|  | Overall, my interaction with the program is very satisfying. | 0.87 |  |

^a^Extraction Method: Principal Component Analysis

^b^Reverse-coded item

**References**

1. Jöns I, Hodapp M, Weiss K. Kurzskala zur Erfassung der Unternehmenskultur. The Leibniz Institute for Psychology (ZPID) 2005. URL: <https://doi.org/10.23668/psycharchives.11386>
2. Nelson LA, Pennings JS, Sommer EC, Popescu F, Barkin SL. A 3-Item Measure of Digital Health Care Literacy: Development and Validation Study. JMIR Form Res 2022;6(4):e36043. PMID:35486413
3. Yamazaki S, Fukuhara S, Green J. Usefulness of five-item and three-item Mental Health Inventories to screen for depressive symptoms in the general population of Japan. Health Qual Life Outcomes 2005;3:48. PMID:16083512
4. Wixom BH, Todd PA. A Theoretical Integration of User Satisfaction and Technology Acceptance. Inf Syst Res 2005;16(1):85-102. doi:10.1287/isre.1050.0042
