# Supplementary material for: Employee Preference and Use of Employee Mental Health Programs: Mixed Methods Study
Source: JMIR Hum Factors. 2025 May 5;12:e65750. doi: 10.2196/65750 (PMC12089874; doi:10.2196/65750)

**Multimedia Appendix 9. Distribution of different types of employee mental health programs (EMHPs) offered by employers in Germany.**


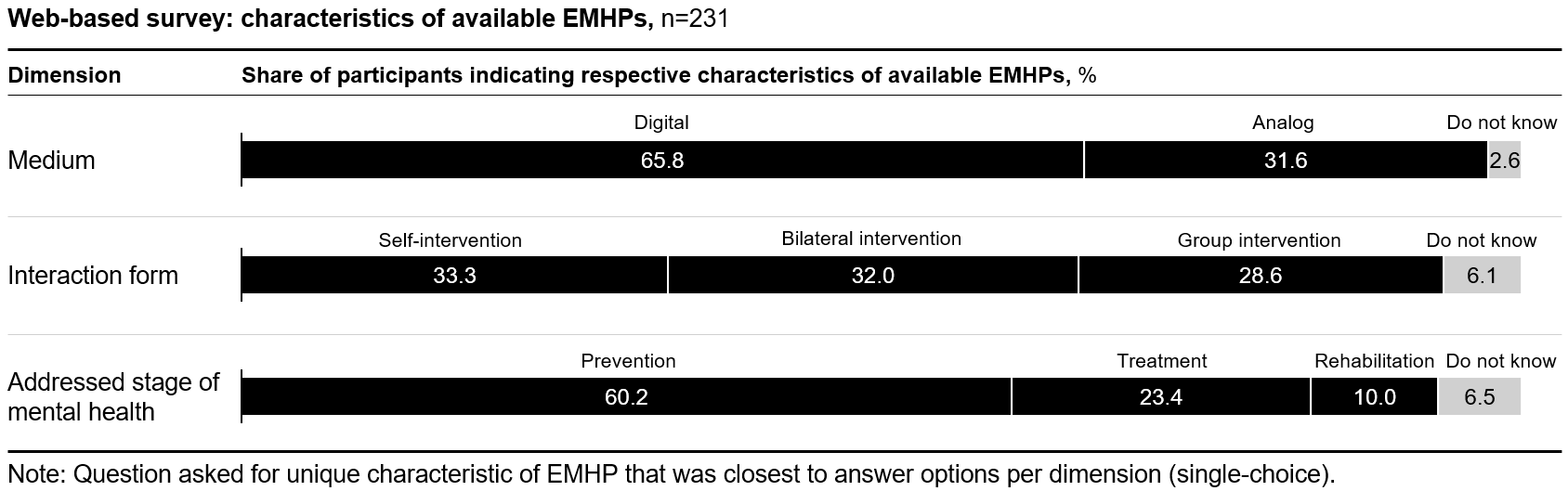

Supplement: Multimedia Appendix 9 [file humanfactors_v12i1e65750_app9.docx]
