# Supplementary material for: Employee Preference and Use of Employee Mental Health Programs: Mixed Methods Study
Source: JMIR Hum Factors. 2025 May 5;12:e65750. doi: 10.2196/65750 (PMC12089874; doi:10.2196/65750)
Supplement: Multimedia Appendix 10 [file humanfactors_v12i1e65750_app10.docx]

**Multimedia Appendix 10. Cross-table of the chi-square test of independence for the relation between the medium of available employee mental health program (EMHP) and actual employee mental health program use.**

|  |  |  | Actual use of EMHP | | |
| --- | --- | --- | --- | --- | --- |
|  |  |  | Yes | No | Total |
| Medium | Digital | n | 94 | 58 | 152 |
|  |  | % | 61.8 | 38.2 | 100.0 |
|  | Analog | n | 32 | 40 | 72 |
|  |  | % | 44.4 | 55.6 | 100.0 |
|  | Employee does not | n | 0 | 6 | 6 |
|  | know | % | 0.0 | 100.0 | 100.0 |
| Total |  | n | 126 | 104 | 230 |
|  |  | % | 54.8 | 45.2 | 100.0 |

X^2^(2, n=230)=13.43, *P*=.001, φ=0.24
