# Supplementary material for: Employee Preference and Use of Employee Mental Health Programs: Mixed Methods Study
Source: JMIR Hum Factors. 2025 May 5;12:e65750. doi: 10.2196/65750 (PMC12089874; doi:10.2196/65750)
Supplement: Multimedia Appendix 11 [file humanfactors_v12i1e65750_app11.docx]

**Multimedia Appendix 11. Cross-table of the chi-square test of independence for the relation between the binary age variable and actual employee mental health program (EMHP) use.**

|  |  |  | Actual use of EMHP | | |
| --- | --- | --- | --- | --- | --- |
|  |  |  | Yes | No | Total |
| Age | Young (≤49 years) | n | 107 | 67 | 174 |
|  |  | % | 61.5 | 38.5 | 100.0 |
|  | Old (≥50 years) | n | 19 | 37 | 56 |
|  |  | % | 33.9 | 66.1 | 100.0 |
| Total |  | n | 126 | 104 | 230 |
|  |  | % | 54.8 | 45.2 | 100.0 |

X^2^(1, n=230)=13.00, *P*<.001, φ=0.24
