# Supplementary material for: Employee Preference and Use of Employee Mental Health Programs: Mixed Methods Study
Source: JMIR Hum Factors. 2025 May 5;12:e65750. doi: 10.2196/65750 (PMC12089874; doi:10.2196/65750)
Supplement: Multimedia Appendix 13 [file humanfactors_v12i1e65750_app13.docx]

**Multimedia Appendix 13. Results of repeated measures ANOVAs analyzing differences in intention to use different employee mental health programs, differentiated by several dimensions.**

| **Dimension** | **Descriptive statistics** | **F-statistics** | | | | | **Effect size** | |
| --- | --- | --- | --- | --- | --- | --- | --- | --- |
| **Medium** | **Mean (SD)** | **df** | **n** | **F** | ***P*** | ***η_p_^2^*** | |  |
| Digital (1) | 4.28 (1.45) | 1 | 1133 | 2.02 | .16 | .002 | |  |
| Analog (2) | 4.23 (1.41) |  |  |  |  |  |  |  |
| *Pairwise comparison^a^* | *Mean difference (SD)* |  |  |  | *p* | *d^d^* | |  |
| 1 vs. 2 | + .04 (0.98) |  |  |  | .16 | 0.04 | |  |
| **Interaction form^b^** | **Mean (SD)** | **df** | **n** | **F** | ***P*** | ***η_p_^2^*** | |  |
| Self-intervention (1) | 4.48 (1.51) | 1.804 | 2044.371 | 134.46 | <.001 | .11 | |  |
| Bilateral intervention (2) | 4.40 (1.48) |  |  |  |  |  |  |  |
| Group intervention (3) | 3.88 (1.66) |  |  |  |  |  |  |  |
| *Pairwise comparison^a^* | *Mean difference (SD)* |  |  |  | *P^c^* | *d^d^* | |  |
| 1 vs. 2 | + .09 (1.14) |  |  |  | .03 | 0.08 | |  |
| 1 vs. 3 | + .60 (1.52) |  |  |  | <.001 | 0.40 | |  |
| 2 vs. 3 | + .51 (1.31) |  |  |  | <.001 | 0.39 | |  |
| **Medium x interaction form^b^** | **Mean (SD)** | **df** | **n** | **F** | ***P*** | ***η_p_^2^*** | |  |
| Digital self-intervention (1) | 4.54 (1.72) | 3.785 | 4288.175 | 79.02 | < .001 | .07 | |  |
| Digital bilateral intervention (2) | 4.40 (1.62) |  |  |  |  |  |  |  |
| Digital group intervention (3) | 3.89 (1.79) |  |  |  |  |  |  |  |
| Analog self-intervention (4) | 4.43 (1.60) |  |  |  |  |  |  |  |
| Analog bilateral intervention (5) | 4.39 (1.60) |  |  |  |  |  |  |  |
| Analog group intervention (6) | 3.88 (1.77) |  |  |  |  |  |  |  |
| *Pairwise comparison^a^* | *Mean difference (SD)* |  |  |  | *P^c^* | *d^d^* | |  |
| 1 vs. 2 | + .14 (1.39) |  |  |  | .015 | 0.10 | |  |
| 1 vs. 3 | + .65 (1.77) |  |  |  | <.001 | 0.37 | |  |
| 1 vs. 4 | + .11 (1.39) |  |  |  | .14 | 0.08 | |  |
| 1 vs. 5 | + .15 (1.70) |  |  |  | .06 | 0.09 | |  |
| 1 vs. 6 | + .66 (1.94) |  |  |  | <.001 | 0.33 | |  |
| 2 vs. 3 | + .52 (1.50) |  |  |  | <.001 | 0.34 | |  |
| 2 vs. 4 | − .03 (1.47) |  |  |  | 1.00 | −0.02 | |  |
| 2 vs. 5 | + .01 (1.28) |  |  |  | 1.00 | 0.01 | |  |
| 2 vs. 6 | + .52 (1.67) |  |  |  | <.001 | 0.31 | |  |
| 3 vs. 4 | − .54 (1.70) |  |  |  | <.001 | −0.32 | |  |
| 3 vs. 5 | − .50 (1.69) |  |  |  | <.001 | −0.30 | |  |
| 3 vs. 6 | + .01 (1.26) |  |  |  | 1.00 | 0.004 | |  |
| 4 vs. 5 | + .04 (1.33) |  |  |  | 1.00 | 0.03 | |  |
| 4 vs. 6 | + .55 (1.68) |  |  |  | <.001 | 0.33 | |  |
| 5 vs. 6 | + .51 (1.47) |  |  |  | <.001 | 0.35 | |  |
| **Addressed stage of mental health^b^** | **Mean (SD)** | **df** | **n** | **F** | ***P*** | ***η_p_^2^*** | |  |
| Prevention (1) | 4.89 (1.61) | 1.879 | 2128.733 | 15.87 | <.001 | .01 | |  |
| Treatment (2) | 4.73 (1.64) |  |  |  |  |  |  |  |
| Rehabilitation (3) | 4.71 (1.62) |  |  |  |  |  |  |  |
| *Pairwise comparison^a^* | *Mean difference (SD)* |  |  |  | *P^c^* | *d^d^* | |  |
| 1 vs. 2 | + .16 (1.21) |  |  |  | <.001 | 0.13 | |  |
| 1 vs. 3 | + .18 (1.26) |  |  |  | <.001 | 0.14 | |  |
| 2 vs. 3 | + .02 (1.01) |  |  |  | 1.00 | 0.02 | |  |

^a^Based on estimated marginal means.

^b^Greenhouse-Geisser correction applied as assumption of sphericity violated.

^c^Bonferroni correction applied for multiple comparisons.

^d^Standard deviation of mean difference used as denominator.
