# Supplementary material for: Employee Preference and Use of Employee Mental Health Programs: Mixed Methods Study
Source: JMIR Hum Factors. 2025 May 5;12:e65750. doi: 10.2196/65750 (PMC12089874; doi:10.2196/65750)
Supplement: Multimedia Appendix 14 [file humanfactors_v12i1e65750_app14.docx]

**Multimedia Appendix 14. Mean comparisons of intention to use different employee mental health programs (EMHPs) between the 2 groups of each binary predicting variable using ANOVA.**

| **Dependent variable** | **Independent (predicting) variable** | | **F-statistics** | | | | | | **Effect size** | | |
| --- | --- | --- | --- | --- | --- | --- | --- | --- | --- | --- | --- |
| **Intention to use** | **Age** | |  |  |  |  | |  | | |  |
| **EMHP type** | **Mean Young (SD)** | **Mean Old (SD)** | **df** | **n** | **F** | ***P*** | ***d*^a^** | | |  |  |
| Overall (all EMHPs) | 4.59 (1.20) | 4.19 (1.40) | 1 | 1131 | 27.76 | <.001 | 0.32 | | |  |  |
| Digital | 4.49 (1.36) | 3.97 (1.53) | 1 | 1131 | 35.87 | <.001 | 0.36 | | |  |  |
| Analog | 4.39 (1.32) | 4.00 (1.51) | 1 | 1131 | 20.89 | <.001 | 0.28 | | |  |  |
| Self-intervention | 4.65 (1.43) | 4.24 (1.59) | 1 | 1131 | 21.02 | <.001 | 0.28 | | |  |  |
| Bilateral intervention | 4.57 (1.39) | 4.14 (1.57) | 1 | 1131 | 23.58 | <.001 | 0.29 | | |  |  |
| Group intervention | 4.09 (1.65) | 3.58 (1.65) | 1 | 1131 | 26.81 | <.001 | 0.31 | | |  |  |
| Prevention | 5.02 (1.54) | 4.68 (1.69) | 1 | 1131 | 12.24 | <.001 | 0.21 | | |  |  |
| Treatment | 4.85 (1.59) | 4.54 (1.71) | 1 | 1131 | 9.88 | .002 | 0.19 | | |  |  |
| Rehabilitation | 4.83 (1.57) | 4.52 (1.69) | 1 | 1131 | 10.03 | .002 | 0.19 | | |  |  |
| **Intention to use** | **Gender** | |  |  |  |  |  | | |  |  |
| **EMHP type** | **Mean Women (SD)** | **Mean Men (SD)** | **df** | **n** | **F** | ***P*** | ***d*^a^** | | |  |  |
| Overall (all EMHPs) | 4.50 (1.24) | 4.37 (1.35) | 1 | 1132 | 2.59 | .11 | 0.10 | | |  |  |
| Digital | 4.33 (1.42) | 4.23 (1.48) | 1 | 1132 | 1.53 | .22 | 0.07 | | |  |  |
| Analog | 4.23 (1.38) | 4.24 (1.45) | 1 | 1132 | 0.02 | .89 | −0.01 | | |  |  |
| Self-intervention | 4.61 (1.49) | 4.38 (1.52) | 1 | 1132 | 7.01 | .008 | 0.16 | | |  |  |
| Bilateral intervention | 4.46 (1.46) | 4.35 (1.49) | 1 | 1132 | 1.51 | .22 | 0.07 | | |  |  |
| Group intervention | 3.78 (1.70) | 3.98 (1.63) | 1 | 1132 | 4.19 | .04 | −0.12 | | |  |  |
| Prevention | 5.06 (1.51) | 4.74 (1.68) | 1 | 1132 | 11.43 | <.001 | 0.20 | | |  |  |
| Treatment | 4.90 (1.58) | 4.57 (1.68) | 1 | 1132 | 11.47 | <.001 | 0.20 | | |  |  |
| Rehabilitation | 4.81 (1.58) | 4.63 (1.65) | 1 | 1132 | 3.54 | .06 | 0.11 | | |  |  |
| **Intention to use** | **Education** | |  |  |  |  |  | | |  |  |
| **EMHP type** | **Mean Academic (SD)** | **Mean Non-academic (SD)** | **df** | **n** | **F** | ***P*** | ***d*^a^** | | |  |  |
| Overall (all EMHPs) | 4.68 (1.24) | 4.26 (1.32) | 1 | 1128 | 28.50 | <.001 | 0.32 | | |  |  |
| Digital | 4.54 (1.41) | 4.10 (146) | 1 | 1128 | 25.67 | <.001 | 0.31 | | |  |  |
| Analog | 4.50 (1.38) | 4.06 (1.41) | 1 | 1128 | 27.42 | <.001 | 0.32 | | |  |  |
| Self-intervention | 4.72 (1.47) | 4.33 (1.51) | 1 | 1128 | 17.76 | <.001 | 0.26 | | |  |  |
| Bilateral intervention | 4.65 (1.43) | 4.23 (1.49) | 1 | 1128 | 22.40 | <.001 | 0.29 | | |  |  |
| Group intervention | 4.20 (1.68) | 3.67 (1.63) | 1 | 1128 | 27.79 | <.001 | 0.32 | | |  |  |
| Prevention | 5.14 (1.52) | 4.72 (1.64) | 1 | 1128 | 18.49 | <.001 | 0.26 | | |  |  |
| Treatment | 4.89 (1.62) | 4.61 (1.66) | 1 | 1128 | 7.89 | .005 | 0.17 | | |  |  |
| Rehabilitation | 4.94 (1.59) | 4.55 (1.64) | 1 | 1128 | 15.44 | <.001 | 0.24 | | |  |  |
| **Intention to use** | **Past mental health issues** | |  |  |  |  |  | | |  |  |
| **EMHP type** | **Mean Past mental issues (SD)** | **Mean No past mental issues (SD)** | **df** | **n** | **F** | ***P*** | ***d*^a^** | | |  |  |
| Overall (all EMHPs) | 4.64 (1.14) | 4.22 (1.41) | 1 | 1105 | 28.97 | <.001 | 0.32 | | |  |  |
| Digital | 4.45 (1.37) | 4.10 (1.52) | 1 | 1105 | 16.39 | <.001 | 0.24 | | |  |  |
| Analog | 4.42 (1.30) | 4.05 (1.51) | 1 | 1105 | 19.06 | <.001 | 0.26 | | |  |  |
| Self-intervention | 4.73 (1.42) | 4.24 (1.55) | 1 | 1105 | 29.80 | <.001 | 0.33 | | |  |  |
| Bilateral intervention | 4.65 (1.37) | 4.15 (1.55) | 1 | 1105 | 32.44 | <.001 | 0.34 | | |  |  |
| Group intervention | 3.93 (1.67) | 3.84 (1.66) | 1 | 1105 | 0.83 | .36 | 0.06 | | |  |  |
| Prevention | 5.15 (1.46) | 4.62 (1.71) | 1 | 1105 | 31.32 | <.001 | 0.34 | | |  |  |
| Treatment | 5.01 (1.52) | 4.43 (1.71) | 1 | 1105 | 35.26 | <.001 | 0.36 | | |  |  |
| Rehabilitation | 4.94 (1.52) | 4.48 (1.70) | 1 | 1105 | 22.27 | <.001 | 0.28 | | |  |  |

| **Intention to use** | **Mental health status** | |  |  |  |  |  |
| --- | --- | --- | --- | --- | --- | --- | --- |
| **EMHP type** | **Mean Positive mental health (SD)** | **Mean Negative mental health (SD)** | **df** | **n** | **F** | ***P*** | ***d*^a^** |
| Overall (all EMHPs) | 4.31 (1.33) | 4.56 (1.21) | 1 | 1113 | 9.92 | .002 | −0.19 |
| Digital | 4.18 (1.45) | 4.38 (1.42) | 1 | 1113 | 5.11 | .02 | −0.14 |
| Analog | 4.12 (1.42) | 4.36 (1.36) | 1 | 1113 | 8.43 | .004 | −0.18 |
| Self-intervention | 4.37 (1.52) | 4.62 (1.44) | 1 | 1113 | 8.14 | .004 | −0.17 |
| Bilateral intervention | 4.29 (1.49) | 4.52 (1.41) | 1 | 1113 | 7.08 | .008 | −0.16 |
| Group intervention | 3.80 (1.64) | 3.97 (1.66) | 1 | 1113 | 2.89 | .09 | −0.10 |
| Prevention | 4.79 (1.67) | 5.00 (1.50) | 1 | 1113 | 4.69 | .03 | −0.13 |
| Treatment | 4.59 (1.70) | 4.89 (1.53) | 1 | 1113 | 9.36 | .002 | −0.19 |
| Rehabilitation | 4.55 (1.68) | 4.91 (1.50) | 1 | 1113 | 13.63 | <.001 | −0.22 |
| **Intention to use** | **Company culture** | |  |  |  |  |  |
| **EMHP type** | **Mean Positive culture (SD)** | **Mean Negative culture (SD)** | **df** | **n** | **F** | ***P*** | ***d*^a^** |
| Overall (all EMHPs) | 4.61 (1.27) | 3.99 (1.27) | 1 | 1132 | 55.48 | <.001 | 0.49 |
| Digital | 4.48 (1.43) | 3.77 (1.38) | 1 | 1132 | 58.32 | <.001 | 0.50 |
| Analog | 4.40 (1.40) | 3.81 (1.36) | 1 | 1132 | 41.69 | <.001 | 0.42 |
| Self-intervention | 4.63 (1.48) | 4.12 (1.52) | 1 | 1132 | 27.91 | <.001 | 0.35 |
| Bilateral intervention | 4.56 (1.44) | 4.00 (1.50) | 1 | 1132 | 34.63 | <.001 | 0.39 |
| Group intervention | 4.13 (1.64) | 3.26 (1.57) | 1 | 1132 | 67.20 | <.001 | 0.54 |
| Prevention | 5.05 (1.51) | 4.47 (1.77) | 1 | 1132 | 31.48 | <.001 | 0.37 |
| Treatment | 4.88 (1.59) | 4.34 (1.72) | 1 | 1132 | 25.56 | <.001 | 0.33 |
| Rehabilitation | 4.87 (1.58) | 4.31 (1.67) | 1 | 1132 | 27.67 | <.001 | 0.35 |

^a^Pooled standard deviation used as denominator
