# Supplementary material for: Employee Preference and Use of Employee Mental Health Programs: Mixed Methods Study
Source: JMIR Hum Factors. 2025 May 5;12:e65750. doi: 10.2196/65750 (PMC12089874; doi:10.2196/65750)
Supplement: Multimedia Appendix 15 [file humanfactors_v12i1e65750_app15.docx]

**Multimedia Appendix 15. Chi-square tests of independence for the relation between employee groups preferring digital or analog employee mental health programs (EMHPs) and perceiving specific facilitators and barriers as relevant for employee mental health program use.**

| **Factor** | **Medium preference group** | |  |  |  |  |  |
| --- | --- | --- | --- | --- | --- | --- | --- |
| **Facilitator** | **Digital preference perceiving as relevant** | **Analog preference perceiving as relevant** | **df** | **N** | **X^2^** | ***P*** | **φ** |
| EMHP is paid for by the employer | 27.78% | 34.19% | 1 | 1134 | 5.34 | .02 | −0.07 |
| EMHP is easy to use | 29.73% | 29.70% | 1 | 1134 | 0.000 | .99 | 0.000 |
| EMHP with trustworthy data protection | 25.23% | 24.36% | 1 | 1134 | 0.11 | .74 | 0.01 |
| EMHP with easy access | 22.97% | 23.93% | 1 | 1134 | 0.14 | .71 | −0.01 |
| EMHP with high content quality | 24.62% | 20.30% | 1 | 1134 | 2.92 | .09 | 0.05 |
| **Barrier** | **Digital preference perceiving as relevant** | **Analog preference perceiving as relevant** | **df** | **N** | **X^2^** | ***P*** | **φ** |
| EMHP requires full payment or copayment by the employee | 37.54% | 39.10% | 1 | 1134 | 0.29 | .59 | −0.02 |
| EMHP requires too much time | 30.33% | 26.50% | 1 | 1134 | 1.97 | .16 | 0.04 |
| EMHP with low content quality | 25.98% | 27.35% | 1 | 1134 | 0.27 | .61 | −0.02 |
| Employees feel forced to use EMHP | 23.12% | 29.91% | 1 | 1134 | 6.60 | .010 | −0.08 |
| EMHP is too complicated or requires too much effort | 22.82% | 24.15% | 1 | 1134 | 0.27 | .60 | −0.02 |
